# Enhancing anti-tumor immunity through co-blocking PD-L1 and TIGIT by facilitating tumor-directed responses and additional VEGF inhibition

**DOI:** 10.3389/fimmu.2025.1746155

**Published:** 2026-01-14

**Authors:** Xi Zhu, Xiaopei Cui, Haijia Yu, Jingen Xu, Xiaofang Chen, Xiaochen Ren, Xiaoyue Wei, Shi Chen, Yangtin Wang, Liyang Fei, Bin Xie, Mingwei Li, Xue Li, Huifeng Jia, Yujie Feng, Simin Xia, Li Chen, Yong Cheng, Lei Zhang, Haidong Li, Xiangyang Zhu, Yifan Zhan

**Affiliations:** 1 Drug Discovery, Shanghai Huaota Biopharmaceutical Co. Ltd., Shanghai, China; 2 Department of Pharmacology, School of Pharmacy, Fudan University, Shanghai, China; 3 Peter MacCallum Department of Oncology & Centre for Cancer Research, University of Melbourne, Melbourne, Australia; 4 College of Biology and Pharmacy, Yulin Normal University, Yulin, China

**Keywords:** bispecific antibody, combination (combined) therapy, PD-(L)1, TIGIT, TME (tumor microenvironment), Treg - regulatory T cell, VEGF

## Abstract

Combination therapy targeting the PD-1/PD-L1 and TIGIT pathways has been explored to enhance the efficacy of current immunotherapies. In this study, we investigated strategies to further potentiate the co-blockade of PD-L1 and TIGIT for cancer immunotherapy. Firstly, we demonstrated that the bispecific antibody (HB0036) for PD-L1 and TIGIT co-blockade induced a greater T-cell proliferative response *in vitro* compared to the combined administration of the parental antibodies. This response was associated with CD226 upregulation and PD-1 downregulation. HB0036 significantly enriched the TIGIT antibody at PD-L1^+^ tumors and achieved improved tumor control with favorable immunological characteristics in both syngeneic and xenograft tumor models. Secondly, we showed that tumor control by co-targeting PD-L1 and TIGIT can be further enhanced by additionally blocking VEGF, a key player in tumorigenesis and tumor angiogenesis, in preclinical studies. Lastly, considering the heterogeneity of tumors, we analyzed how the expression patterns of PD-L1 and CD155 influence T cell responses. We also examined the spatial distribution of PD-L1 and CD155, along with related immunological parameters from patient samples, to assess the potential of PD-L1 and TIGIT co-blockade in diverse tumor contexts.

## Highlights

- The bispecific PD-L1 and TIGIT co-blockade demonstrated superior immune restoration *in vitro* compared to the combination of the two parental monoclonal antibodies.
- The bispecific antibody preferentially enriched higher levels of TIGIT antibody accumulation in PD-L1^+^ tumors.
- Tumor control was significantly improved with the bispecific antibody compared to combined administration of the parental antibodies.
- The efficacy of PD-L1 and TIGIT co-blockade could be further enhanced through additional VEGF inhibition.
- Heterogeneous expression patterns of PD-L1 and CD155, along with associated immunological parameters, may have important clinical implications for PD-L1 and TIGIT co-targeting strategies.

## Introduction

Recent studies have highlighted the potential of PD-1 and TIGIT co-blockade to enhance T-cell responses and inhibit tumor growth (1, 2). However, the clinical benefits of using two antibodies for co-blockade, compared to anti-PD-L1 monotherapy, remain inconclusive across various clinical studies, including those focusing on non-small cell lung cancer (NSCLC) (3–5). This underscores the need for further exploration of pathways that can enhance the efficacy of PD-1 and TIGIT co-blockade.

A promising approach within combination therapies involves the use of bispecific antibodies (BsAbs). These engineered molecules possess two distinct arms, each capable of recognizing different epitopes or molecules, thereby facilitating cell–cell and protein–protein interactions to improve therapeutic efficacy (6, 7). Several BsAbs targeting PD-(L)1 and TIGIT are currently progressing through clinical trials, including the PD-1 targeting BsAb Rilvegostomig (8), ZG005 (9), and IBI321 [NCT04911881] (10). Additionally, PD-L1 targeting antibodies such as PM1022 (11), HB0036 (12), and HLX301 [NCT05102214] (10) are also in development.

Despite these advancements, and while several groups have reported the development of PD-L1/TIGIT BsAbs with potent anti-tumor activity (13–16), a significant knowledge gap remains. It is not fully understood whether the bispecific format offers mechanistically distinct advantages over the co-administration of two individual antibodies. For example, it is unclear how a BsAb might uniquely modulate the interplay between inhibitory (TIGIT, PD-1) and co-stimulatory (CD226) receptors on T cells, or how its efficacy is determined by the spatial arrangement of PD-L1 and TIGIT ligands in the tumor microenvironment. In this study, we used the anti-PD-L1/TIGIT bispecific antibody HB0036 to investigate these precise questions, providing cellular and molecular evidence for the unique advantages a BsAb may offer in enhancing anti-tumor immunity.

Crucially, HB0036 was engineered with an active Fc region, based on the rationale that TIGIT blockade relies partially on Fc-mediated depletion of regulatory T cells (Tregs) for maximal efficacy, a feature lacking in Fc-silent variants. This strategy of leveraging a PD-L1 backbone to counteract Treg-mediated immunosuppression parallels the mechanistic logic of other bispecific platforms, such as YM101 (anti-PD-L1/TGF-β). While YM101 restores immunity by inhibiting TGF-β-induced Treg differentiation (17, 18), HB0036 is designed to physically deplete intratumoral Tregs via ADCC, thereby reinvigorating anti-tumor responses.

To extend the therapeutic window and improve the efficacy of PD-1/PD-L1 and TIGIT co-blockade, various combination strategies with other anti-cancer agents have been explored. For instance, combining chemotherapy agents like docetaxel with the A2R antagonist etrumadenant has been tested (19). The anti-PD-1/TIGIT bispecific antibody Rilvegostomig has also been combined with datopotamab deruxtecan for metastatic non-small cell lung cancer (TROPION-Lung10) [NCT06357533]. Notably, combining PD-(L)1 and TIGIT co-blockade with VEGF-targeting antibodies has shown promising results in treating advanced hepatocellular carcinoma (20). Given the recent focus on BsAbs targeting PD-(L)1 and VEGF as next-generation immunotherapies (21), we investigated tumor inhibition through two combination approaches: anti-PD-L1/VEGF BsAb HB0025 (22) with anti-TIGIT antibody HB0030 (23), and HB0036 with VEGF-neutralizing antibody 2.1T (22).

Finally, since PD-L1 is expressed on both tumor cells and tumor-infiltrating immune cells, durable responses to atezolizumab (anti–PD-L1) have been observed in patients with high PD-L1 expression on either tumor cells or immune cells alone (24). Complicating matters further, the clinical efficacy of PD-(L)1 blockade appears to depend on the co-expression patterns of PD-L1 and CD155 (PVR), the ligand for TIGIT and a costimulatory receptor for CD226 (25). This raises an important question: who is most likely to benefit from PD-(L)1 and TIGIT co-blockade? To address this, we examined the expression patterns of PD-L1 and CD155 in patient samples and tumor cell lines derived from both mouse and human sources. Using *in vitro* culture systems, we further explored how the expression levels of PD-L1 and CD155 influence T-cell immunity and modulate the efficacy of PD-1/PD-L1 and TIGIT co-blockade.

## Results

### PD-L1 and TIGIT targeting bispecific antibody HB0036 is more potent in reversing immune inhibition than combination of two parental mabs

**Figure 1:**
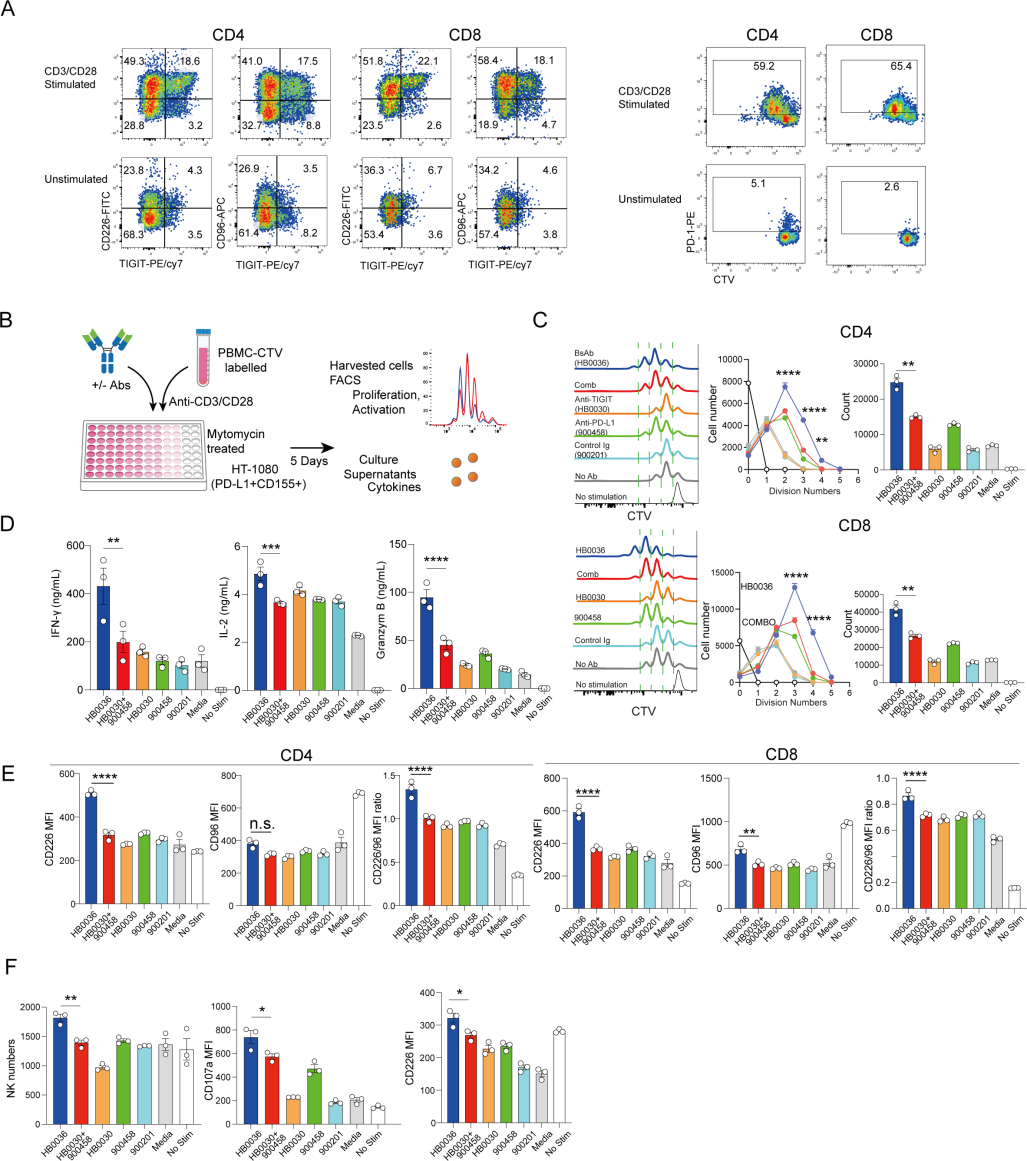
PD-L1 and TIGIT targeting bispecific antibody HB0036 is more potent in reversing immune inhibition than combination of two parental mAbs: **(A)** Activation induced expression of co-receptors: Human PBMCs were stimulated *in vitro* with mitogenic antibodies against CD3 and CD28. Harvested cells were stained for surface expression of the indicated markers. FACS plots show expression of CD226, TIGIT, CD96 and PD-1. **(B–F)** Enhancement of T cell responses *in vitro*. **(B)** CTV-labelled PBMCs were cultured with mitomycin-treated HT-1080 cells in 96-well plates with indicated Abs at equal molar concentrations. Cells were then stimulated with mitogenic antibodies against CD3 and CD28 for 5 days. Cell proliferation, expression of surface markers and cytokine production were determined. **(C)** Histograms display cell division; line graphs show cell numbers at given cell divisions and bar graphs show total recovered proliferating T cells. **(D)** Bar graphs show cytokine levels from culture supernatants. **(E)** Bar graphs show the expression (mean MFI ± SEM) of CD226, CD96 and CD226 MFI/CD96 MFI ratio by CD4^+^ and CD8^+^ T cells. **(F)** Bar graphs show recovered NK cell numbers, expression of CD107a and CD226 by NK cells. *p < 0.05, **p < 0.01, ***p < 0.001, ****p < 0.0001 by One-way ANOVA multiple parameter comparison, n.s., not significant.

The bispecific antibody HB0036, targeting both PD-L1 and TIGIT, is currently undergoing clinical evaluation (12). HB0036 was engineered from a humanized anti-PD-L1 antibody previously described (22) and a humanized anti-TIGIT antibody HB0030 (CTR20212828, patent No. 202010387630.4). It was constructed by linking tandem anti-TIGIT single-chain variable fragments (scFvs) of HB0030 to the C-terminus of the heavy chain of a humanized anti-PD-L1 IgG1, resulting in an IgG-scFv format (**Supplementary Figures 1A–C**). The selected HB0036 bispecific antibody retained high affinity (**Supplementary Figures 1D–F**) and functional activity (**Supplementary Figures 1G–I**) comparable to the parental monoclonal antibodies. Under stimulation with anti-CD3/CD28 antibodies, both CD4^+^ and CD8^+^ T cells showed increased expression of the inhibitory receptor TIGIT, the stimulatory receptor CD226, and the inhibitory receptor CD96 (**Figure 1A**). Similarly, TCR stimulation led to the upregulation of PD-1, particularly on proliferating T cells (**Figure 1A**).

Next, we compared the immune-restorative capabilities of the bispecific antibody HB0036 with those of combinations of the parental monoclonal antibodies anti-PD-L1 (900458) and anti-TIGIT (HB0030), using an *in vitro* culture system with human peripheral blood mononuclear cells (PBMCs). To mimic the tumor microenvironment, human PBMCs were stimulated with anti-CD3/CD28 antibodies in the presence of CD155^+^ and PD-L1^+^ tumor cells (HB1080) (**Figure 1B**). Under these conditions, both CD4^+^ and CD8^+^ T cells proliferated, achieving greater expansion compared to unstimulated cultures (**Figure 1C**). The addition of anti-PD-L1 900458, a mixture of equimolar amounts of anti-PD-L1 and anti-TIGIT monoclonal antibodies, as well as HB0036, significantly enhanced T cell proliferation. Notably, HB0036 induced stronger proliferative responses and cytokine production compared to the combination treatment (**Figures 1C, D**, **Supplementary Figure 2A**). The proportions of IFN-γ-producing T cells did not differ significantly among the groups, indicating that the enhanced proliferation primarily drove cytokine output (**Supplementary Figure 2B**).

Given the critical role of the co-activating receptor CD226 in augmenting anti-tumor immunity via TIGIT or PD-1/PD-L1 blockade (2), we analyzed the expression of CD226 and the inhibitory receptor CD96 on T cells under various conditions. Our results showed that HB0036, unlike the combination treatment, increased the expression of CD226 on both CD4^+^ and CD8^+^ T cells (**Figure 1E**, **Supplementary Figure 2C**). While CD96 was moderately upregulated on CD8^+^ T cells, the ratio of CD226 to CD96 remained higher in cultures treated with HB0036 (**Figure 1E**).

Additionally, HB0036 treatment increased natural killer (NK) cell numbers and activation, as indicated by CD107a expression (**Figure 1F**). The proportion of IFN-γ-producing NK cells was also elevated in the HB0036 group (**Supplementary Figure 2D**). Notably, while anti-TIGIT antibody HB0030 alone did not significantly affect T cell proliferation, it did enhance CD107a expression on NK cells (**Supplementary Figure 2E**).

### HB0036 enhances intratumoral accumulation of anti-TIGIT antibody via engagement of the anti-PD-L1 arm

**Figure 2:**
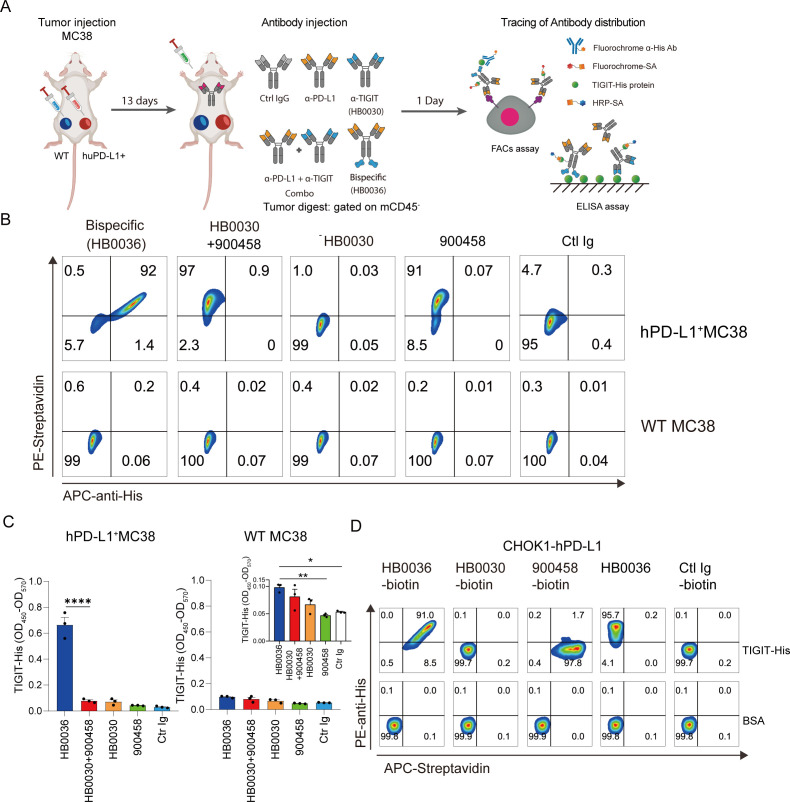
HB0036 enhances intratumoral accumulation of Anti-TIGIT antibody via PD-L1 engagement. **(A)** Experimental Design for Assessing Intratumoral Antibody Distribution>: hTIGIT/hPD-L1/hPD-1 humanized C57BL/6 mice were inoculated with hPD-L1^+^ and hPD-L1^-^ WT MC38 cells on contralateral flanks. After 13 days, biotinylated antibodies were administered, and tumors were harvested 24 hours later for analysis of antibody accumulation. **(B)***In Vivo* Antibody Binding to Tumor Cells. Tumor digests were incubated with TIGIT-His protein, then stained with PE-Streptavidin (to detect biotinylated antibodies), APC anti-His (to identify anti-TIGIT antibodies), and mCD45. Flow cytometry plots show antibody binding to tumor cells (gated on live mCD45^-^ cells). **(C)** Quantification of TIGIT Antibody Accumulation in Tumors. Total TIGIT antibody levels in tumor digests were measured by ELISA. **(D)***In Vitro* Antibody Binding Assay. CHOK1-hPD-L1 cells were incubated with equimolar concentrations of biotinylated HB0036, HB0030, 900458, control IgG, or biotin-free HB0036 for 30 min on ice. Cells were then treated with His-tagged human TIGIT protein, followed by staining with PE-Streptavidin and APC anti-His antibodies. *p < 0.05; **p < 0.01; ****p < 0.0001.

A key advantage of bispecific antibodies (BsAbs) is their ability to bridge immune cells to tumor cells by targeting tumor-expressing molecules. PD-L1 is upregulated within the tumor microenvironment (TME) on both tumor cells and infiltrating leukocytes. Here, we compared drug accumulation within tumors. Using humanized C57BL/6 mice (*n=3* per group), inoculated subcutaneously (*s.c.*) with 1×10^6^ wild-type hPD-L1 MC38 cells on the left back and 10^6^ hPD-L1^+^ MC38 cells on the right, we allowed tumors to grow to approximately 200 mm³ (13 days post-inoculation). Mice were then injected intraperitoneally with either biotinylated HB0036 (683 μg, equimolar to other groups), anti-TIGIT HB0030, anti-PD-L1 900458, a combination of HB0030 and 900458, or control IgG 900201 (all 500 μg). Tumors were harvested 24 hours post-injection for analysis of antibody distribution and immune cell infiltration (**Figure 2A**). Analysis of live CD45^–^ tumor cells stained for antibody binding revealed that HB0036 exhibited abundant binding to both anti-PD-L1 (detected via PE-streptavidin) and anti-TIGIT (detected via His-tagged TIGIT protein with APC-anti-His antibody) on hPD-L1^+^ MC38 cells. In contrast, co-injection of individual antibodies showed strong binding only for anti-PD-L1, with negligible anti-TIGIT binding (**Figure 2B**). Importantly, minimal antibody binding was observed across all groups in hPD-L1^–^ MC38 tumor cells (**Figure 2B**).

Quantification of anti-TIGIT antibody accumulation within tumor lysates by ELISA demonstrated substantial accumulation of anti-TIGIT in hPD-L1^+^ MC38 tumors injected with HB0036 (**Figure 2C**). Notably, HB0036 also resulted in detectable, albeit lower, levels of anti-TIGIT antibody in hPD-L1^–^ MC38 tumors, likely through engagement of PD-L1 expressed on immune cells (**Figure 2C**, inset). This enhanced binding of HB0036 to PD-L1^+^ tumor cells was further confirmed *in vitro* using CHOK1 cells expressing human PD-L1 incubated with various antibodies (**Figure 2D**).

### HB0036 with ADCC capacity reduces Tregs *in vitro* and *in vivo*

It has been reported that anti-TIGIT antibodies decrease the frequency of intratumoral regulatory T cells (Tregs) via Fc-dependent cytotoxicity (ADCC) (26). To investigate the role of ADCC in TIGIT-blocking antibodies, we introduced mutations at leucines 234 and 235 (EU numbering) in the CH2 region of the HB0030 Fc domain, substituting them with alanines (L234A/L235A). This mutation abolishes the antibody’s binding affinity to Fcγ receptors and C1q, thereby eliminating its ADCC and complement-dependent cytotoxicity (CDC) activities. We then utilized these variants to evaluate the specific contribution of Fc-mediated effector functions, starting with *in vitro* cytotoxicity assays.

**Figure 3:**
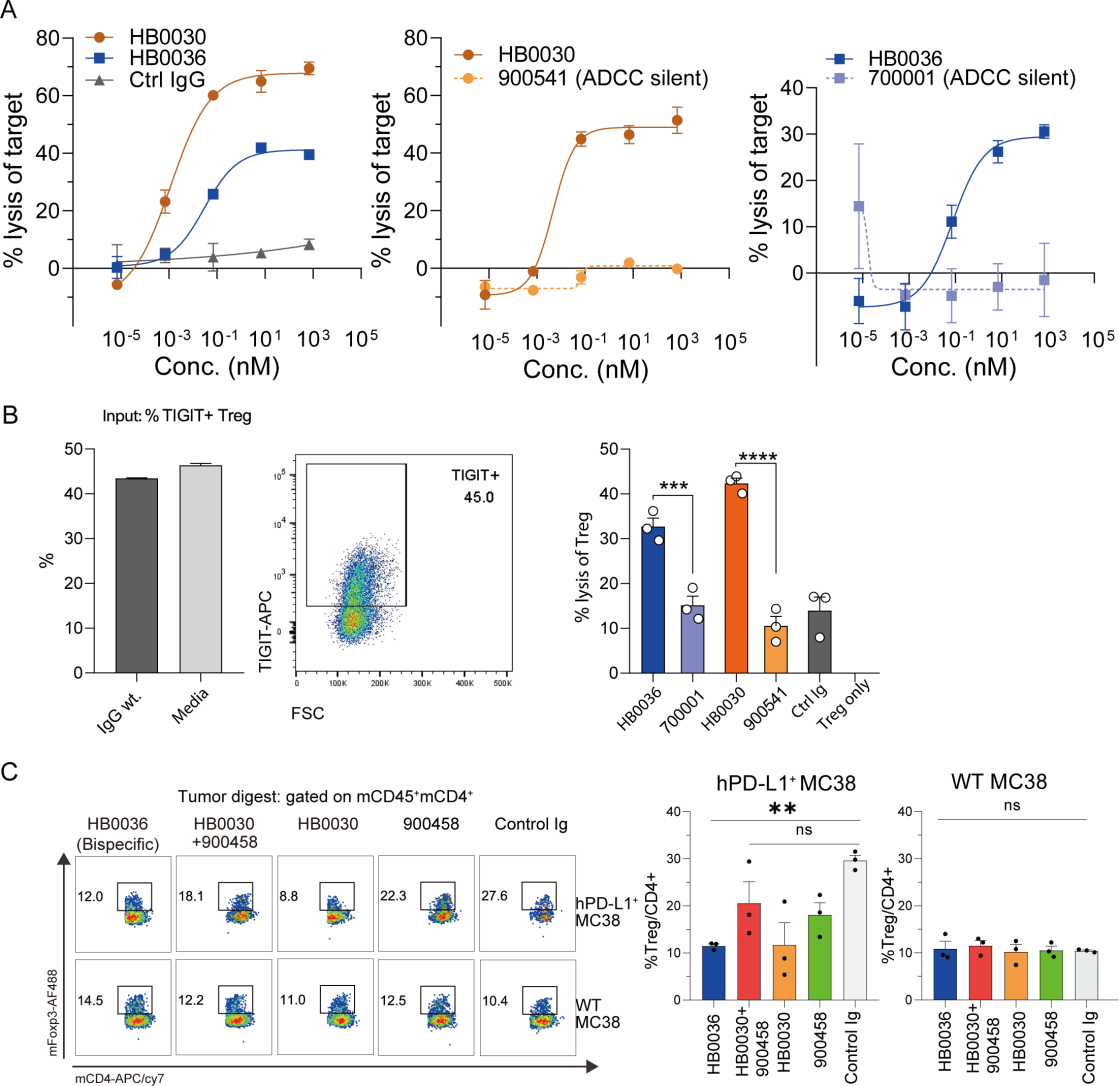
HB0036 with ADCC capability reduces Tregs *in vitro* and *in vivo*. **(A)** NK cell-mediated target cell killing. Human NK cells (1×10^5^/well) were co-cultured with Jurkat-TIGIT-22G8 target cells (1×10^5^/well) in 96-well U-bottom plates for 5 hours in the presence of serially diluted antibodies. Line graph depicts Jurkat cell lysis (%). **(B)** NK cell-mediated Treg killing. Tregs (~40% TIGIT^+^) were seeded at 5×10^4^ cells/well in 96-well round-bottom plates and treated with indicated antibodies: isotype control (wild-type Fc), HB0030, HB0036, HB0030 KO (ADCC-silent, #900541), or HB0036 KO (ADCC-silent, #700001). Bar graph shows Treg lysis (%). ***p < 0.001, ****p < 0.0001 (one-way ANOVA). **(C)***In vivo* Treg reduction. Experimental setup as in **Figure 2**. Humanized mice bearing contralateral hPD-L1^+^/hPD-L1^-^ MC38 tumors were treated with biotinylated antibodies for 1 day after 13 days of tumor growth. Tumor-infiltrating mCD45^+^ cells were analyzed for CD4^+^FoxP3^+^ Tregs. FACS plots show Tregs within CD4^+^ cells; bar graphs depict Treg frequency (% of CD4^+^ cells, mean ± SEM). **p < 0.01, ns (not significant; one-way ANOVA).

We assessed the capacity of TIGIT blockade to facilitate NK cell-mediated killing of TIGIT^+^ cells. HB0036 demonstrated NK-dependent killing of TIGIT-expressing cells *in vitro* (**Figure 3A**). However, the cytotoxic activity of HB0036 against huTIGIT-expressing Jurkat cells was weaker compared to the ADCC-competent anti-TIGIT antibody HB0030 (**Figure 3A**).

Next, we examined the *in vitro* killing of Treg cells by NK cells, with or without TIGIT-blocking antibodies. Isolated Tregs from PBMCs comprised approximately 50% TIGIT^+^ cells (**Figure 3B**). In co-culture assays, both HB0036 and HB0030 with intact ADCC activity significantly increased Treg cell killing (**Figure 3B**). Conversely, the ADCC-silent variants of HB0036 and HB0030 showed markedly reduced cytotoxicity (**Figure 3B**). Consistent with earlier findings, HB0030 exhibited stronger Treg killing than HB0036 (**Figure 3B**).

We then evaluated the impact of these therapeutic antibodies on intratumoral Tregs in an *in vivo* model. Using the approach outlined in **Figure 2A**, humanized BABL/c mice expressing hTIGIT/hPD-L1/hPD-1 were subcutaneously inoculated with 1×10^6^ wild-type hPD-L1-MC38 cells on the left flank and 1×10^6^ hPD-L1^+^ MC38 cells on the right. Once tumors reached approximately 200 mm³, mice received intraperitoneal injections of the antibodies. Tumors were harvested 24 hours post-injection for immune infiltrate analysis. No significant changes were observed in the total CD4^+^ T cell populations within the tumors of PD-1/PD-L1 humanized mice (**Supplementary Figure 3B**). Notably, hPD-L1^+^ MC38 tumors exhibited a higher percentage of FoxP3^+^ Tregs compared to hPD-L1^-^ MC38 tumors (**Figure 3C**), reaffirming PD-L1’s role in Treg development and maintenance (27, 28).

Treatment with both HB0036 and HB0030 significantly reduced the proportion of FoxP3^+^ Tregs in tumor-infiltrating lymphocytes (TILs) (**Figure 3C**), although HB0036 showed weaker Treg depletion *in vitro* compared to HB0030 (**Figure 3B**). Interestingly, the combination of HB0030 with 900458 did not result in a clear reduction in Treg levels, likely due to the Fc fragment of anti-PD-L1 could also bind to effector cells, lowering the probability of TIGIT antibody engagement and thus resulting in decreased depletion of Tregs. In hPD-L1^−^ MC38 tumors, which harbor a lower Treg frequency, none of the treatments significantly impacted Treg abundance.

### HB0036 exhibits a stronger anti-tumor effect than combination therapy in two preclinical models

We first investigated the effects of the anti-TIGIT antibody HB0030, the anti-PD-L1 antibody 900458, and the combination of HB0030 with anti-PD1 or anti-PD-L1 antibodies on tumor growth. Using a subcutaneous model of the murine colorectal carcinoma cell line CT26 in hTIGIT/hPD-1 BALB/c mice, we found that HB0030 with ADCC function had strong tumor inhibition compared to its ADCC-silent counterpart (**Supplementary Figure 3A**). In the subcutaneously implanted CT26-hPD-L1 in the hPD-1/hPD-L1 BALB/c mouse model, the anti-PD-L1 antibody 900458 also exhibited tumor inhibition (**Supplementary Figure 3C**). To investigate the anti-tumor effects of PD-L1 and TIGIT co-blockade, hPD-1/hPD-L1/hTIGIT humanized C57BL/6 mice were injected with hPD-L1-MC38 cells. Combination of HB0030 and anti-PD-L1–900458 showed better tumor control relative to single agents (**Supplementary Figure 3D**).

**Figure 4:**
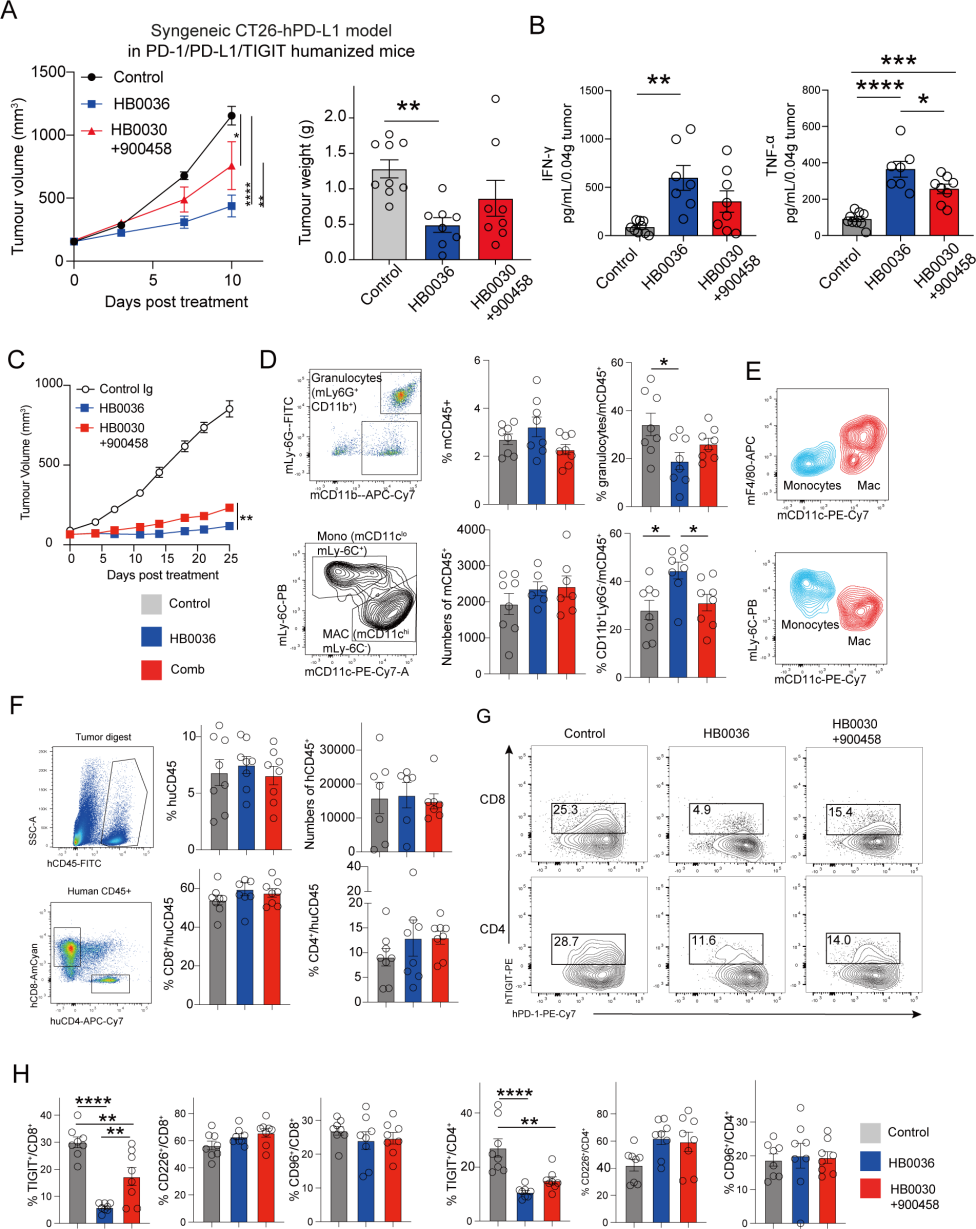
HB0036 Exhibits a stronger anti-tumor effect than combination therapy in two preclinical models. hPD1/hPDL1/hTIGIT BALB/c mice were inoculated with 1×10^6^ CT26-hPDL1 cells on the flank. Treatment (*n=8-9*) was started when tumour sizes were an average TV of 160 mm^3^. Equimolar dose of Abs (13.7 mg/kg body weight HB0036 or 10 mg/kg 900458 and 10 mg/kg body weight HB0030, twice a week, total 4 doses) were given intraperitoneally. At the end of the experiment, tumour weights were recorded. Tumours were digested for TIL analysis. **(A)** Tumor volumes and tumour weights. P values for tumour volumes were calculated with Prism two-way ANOVA Tukey’s multiple-comparisons test. P values for tumour weights were generated with one-way ANOVA multiple multiple-comparisons test. *p < 0.05, **p < 0.01, ***p < 0.001, ****p < 0.0001. **(B)** tumoral cytokine content. *p < 0.05, **p < 0.01, ***p < 0.001, **** *p* < 0.0001 by one-way ANOVA multiple multiple-comparisons test. **(C–H)** BxPC-3 (1×10^7^ cells) were inoculated subcutaneously into NPG mice on the right flank. When tumours had grown to an average size of 80 mm^3^, tumour-bearing mice were divided into 4 groups (*n=8* each) and injected *i.v.* with 1×10^7^ PBMCs. Treatments started on the same day and antibodies were dosed twice weekly for 4 weeks intraperitoneally. **(C)** Tumour volume. **p < 0.01 by two-way ANOVA Tukey’s multiple-comparisons test. **(D, E)**. mouse myeloid TILs: Analysis of FACS plots show the gating of mouse myeloid cells within mouse CD45^+^ TILs. **(F–H)** human lymphocytic TILs: **(F)** The percentages and the numbers of human TILs, **(G)** FACS plots show the expression of TIGIT and PD-1 by human CD8^+^ and CD4^+^ T cells. **(H)** Bar graphs show the percentages of human T-cell subsets with expression of TIGIT, CD226 or CD96. *p < 0.05, **p < 0.01, ****p < 0.0001 by one-way ANOVA multiple-comparisons test.

Based on both *in vitro* and *in vivo* data suggesting that bispecific antibodies (BsAbs) may offer advantages over combination therapies in certain settings, we evaluated tumor control using HB0036 and a combination regimen. Tumor inhibition was initially assessed in a syngeneic mouse model with humanized hTIGIT/hPD-1/hPD-L1. To capture early immunologic changes before tumor volume became excessive, tumors were harvested earlier. After 10 days of treatment in the hPD-L1 CT26 syngeneic carcinoma model, we observed significantly reduced tumor volume and weight in the HB0036 group (**Figure 4A**). Among the immunological parameters correlating with tumor inhibition, levels of IFN-γ and TNF-α were notably higher in the HB0036-treated group (**Figure 4B**). TILs and CD8^+^ TILs, normalized to the same tumor weight, were more abundant in both the HB0036 and combination groups compared to controls, but no significant differences were observed between the two (**Supplementary Figures S4A, B**). Notably, the number of CD4^+^ T cells was slightly reduced in the HB0036 group without a further specific decrease in Tregs (**Supplementary Figures S4C, D**). The CD226^+^ T cell population was slightly elevated in both the HB0036 and combination groups (**Supplementary Figure 4E**). In the myeloid compartment, no significant changes were detected (**Supplementary Figure 4F**). These findings suggest that HB0036 confers subtle immunological advantages over combination therapy in promoting anti-tumor responses. Subsequently, we compared tumor control by HB0036 versus combination therapy in a subcutaneous BxPC-3 xenograft model using human PBMC-humanized Prkdc^scid^/Il2rg^null^ (NPG) mice. Both treatments achieved good tumor suppression compared to controls, with HB0036 showing slightly stronger inhibition than the combination, although the differences were modest (**Figure 4C**). In this model, tumor-infiltrating myeloid cells were of mouse origin, while lymphocytes were human, derived from transferred PBMCs. Mouse CD11b^+^ myeloid cells were divided into two main subsets: Ly6G^+^ neutrophils and Ly6G^−^ cells (**Figure 4D**). Notably, the HB0036 group exhibited more Ly6G^−^ CD11b^+^ cells and fewer Ly6G^+^ CD11b^+^ cells compared to controls and the combination group (**Figure 4D**). The Ly6G^−^ subset included Ly6C^+^ monocytes and F4/80^+^ macrophages (**Figure 4D**), with no significant differences in their relative abundance across the groups. Human T cells within TILs comprised CD8^+^ and CD4^+^ populations (**Figure 4F**). While total T-cell abundance remained similar across groups, the proportion of TIGIT^+^ T cells was significantly reduced in the HB0036 group compared to controls and the combination group, with no significant changes observed in CD226^+^ or CD96^+^ T cells (**Figures 4G, H**). In addition, enhanced tumor inhibition was also observed in xenograft models using the human bladder carcinoma cell line HT1367 in PBMC-humanized mice (**Supplementary Figure 4G**). Overall, HB0036 achieved better tumor control, accompanied by some immunological differences.

### Triple blockade of PD-L1, TIGIT, and VEGF enhances anti-tumor efficacy in preclinical models

**Figure 5:**
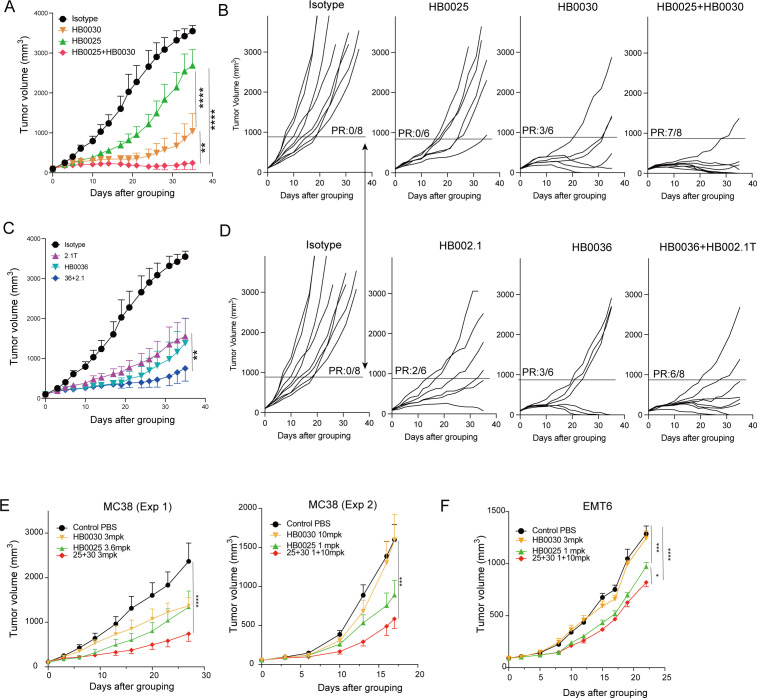
Triple blockade of PD-L1, TIGIT, and VEGF enhances anti-tumor efficacy in preclinical models. **(A–D)** H22-hPD-L1 tumor model in hPD-1/hPD-L1/hTIGIT BALB/c mice: Mice (n=6–8/group) were inoculated with 1×10^6^ H22-hPD-L1 cells and treated at an average tumor volume (TV) of 100 mm³. Treatment groups: isotype control (10 mg/kg), HB0036 (4.1 mg/kg), 2.1T (VEGF TRAP, 1 mg/kg), HB0036 + 2.1T, HB0025 (anti-PD-L1/VEGF, 2 mg/kg), HB0030 (anti-TIGIT mAb, 3 mg/kg), or HB0025 + HB0030. Antibodies were administered intraperitoneally (*i.p.*) twice weekly (4 total doses). **(A, B)** Tumor volumes and partial responses (defined as tumor volume reductions exceeding two-thirds of the mean tumor volume in the isotype control group at the final measurement) for isotype, HB0025, HB0030, and HB0025 + HB0030. **(C, D)** Tumor volumes and partial responses for isotype, HB0036, 2.1T, and HB0036 + 2.1T. **(E)** hPD-L1 MC-38 tumor model in hTIGIT/hPD-L1/hPD-1 humanized C57BL/6 mice: Mice (*n=6*/group) were inoculated with 1×10^6^ hPD-L1-expressing MC-38 cells and treated at TV ≈100 mm³. Groups: PBS, HB0025 (3.6 mg/kg), HB0030 (3 mg/kg), or combination. Antibodies were given IP twice weekly (4 doses). Tumor volumes shown. **(F)** EMT6-hPD-L1 model in hPD-1/hPD-L1/hTIGIT BALB/c mice: Mice (n=6/group) were inoculated with 2×10^6^ hPD-L1-expressing EMT6 and treated at TV ≈100 mm³. Treatment groups: isotype (3 mg/kg), HB0025 (1 mg/kg), HB0030 (3 mg/kg), or combination. Tumor volumes shown. Statistical significance: *p < 0.05, **p < 0.01, ***p < 0.001, ****p < 0.0001 (two-way ANOVA, Tukey’s multiple comparisons test).

Combined blockade of PD-(L)1, TIGIT, and VEGF has shown promising results in improving clinical outcomes for patients with unresectable, locally advanced, or metastatic hepatocellular carcinoma (HCC) (20). Here, we investigated the therapeutic potential of this triple blockade in preclinical models. Most experiments were conducted using the anti-PD-L1/VEGF bispecific antibody HB0025 and anti-TIGIT Ab HB0030. In hPD1/hPDL1/hTIGIT BALB/c mice bearing huPD-L1^+^ H22 tumors, treatment efficacy was assessed based on mean tumor volume and partial responses (defined as tumor volume reductions exceeding two-thirds of the mean tumor volume in the isotype control group at the final measurement). Both HB0025 (PD-L1/VEGF bispecific Ab), HB0030 (anti-TIGIT), and their combination demonstrated significant tumor inhibition compared to the isotype control (**Figures 5A, B**). Notably, the combination therapy exhibited superior tumor suppression compared to monotherapy with either agent (**Figures 5A, B**). We further evaluated the triple blockade using HB0036 (anti-PD-L1/TIGIT bsAb) combined with the VEGF inhibitor HB002.1T (22, 29). Similar to the previous findings, HB0036, HB002.1T, and their combination all showed significant tumor inhibition versus the control (**Figures 5C, D**). While the combination trended toward stronger efficacy than HB002.1T monotherapy, statistical significance was not reached due to intragroup variability in HB0036-treated mice. Importantly, the triple blockade did not induce body weight changes, suggesting a favorable safety profile (**Supplementary Figure 4H**). Additional validation in three independent models further confirmed the superior anti-tumor activity of the triple blockade (**Figures 5E, F**). Thus, despite variations in tumor models and dosing regimens, the triple blockade consistently outperformed dual blockade achieve with BsAbs.

### The superior efficacy of bispecific antibody HB0036 requires co-expression of PD-L1 and CD155 on the same target cell

**Figure 6:**
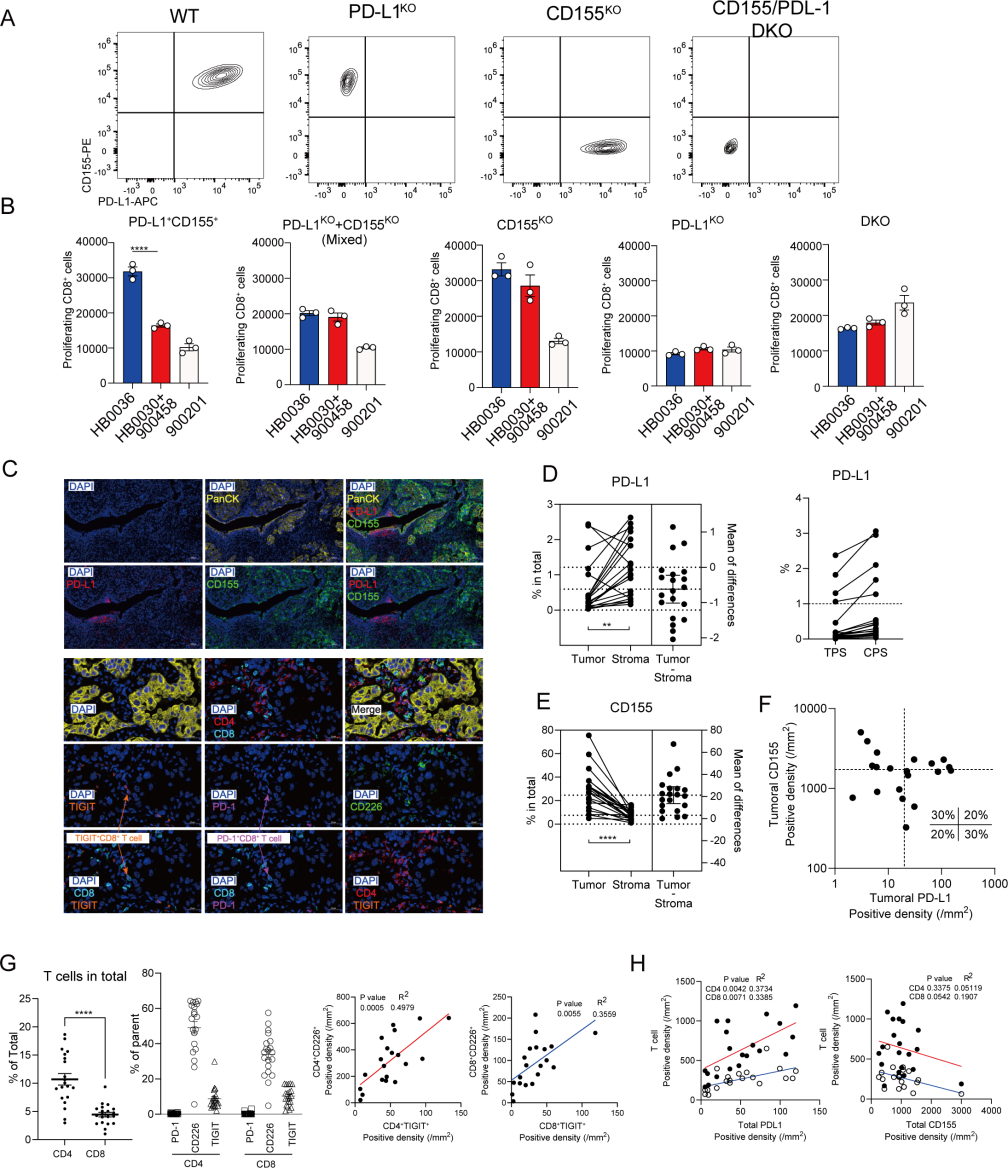
Pattern of expression of PD-L1 and CD155 (PVR) and its correlation to blocking and immune landscape. **(A)** FACS plots show expression pattern of PD-L1 and CD155 by HT-1080 cells. **(B)** CTV-labelled PBMCs were cultured with mitomycin-treated HT-1080 cells in 96-well plates with indicated Abs at equal molar concentrations. Histograms show the numbers of proliferated cells under stimulated conditions. ****p < 0.0001 by one-way ANOVA multiple-comparisons test. **(C–H).** High-Content Analysis of human lung adenocarcinoma microenvironment. Multiplex immunofluorescence and quantitative analysis were used to profile the tumor (PanCK^+^) and stromal (PanCK^-^) compartments. **(C)** Representative High-Content analysis images illustrating the immune landscape in adenocarcinoma tissue. **(D)** Quantification of the percentage of cells expressing PD-L1 within the PanCK^+^ tumor and PanCK^-^ stromal compartments. Lines connect paired tumor and stroma samples from the same patient. The mean difference between compartments is plotted on the right axis for each graph. PD-L1 expression was then further expressed as Tumor Proportion Score (TPS), calculated as the ratio of PanCK^+^ PD-L1^+^ cells to PanCK^+^ tumor cells, and a Combined Positive Score (CPS), calculated as the ratio of all PD-L1^+^ cells to PanCK^+^ tumor cells. **(E)** Quantification of the percentage of cells expressing CD155 within the PanCK^+^ tumor and PanCK^-^ stromal compartments. Lines connect paired tumor and stroma samples from the same patient. The mean difference between compartments is plotted on the right axis for each graph. **(F)** Co-expression analysis of PD-L1 and CD155 on PanCK^+^ tumor cells. Each dot represents a patient sample. Dashed lines indicate the median density of the cohort for each marker, stratifying tumors into four subsets. **(G)** Graphs show Frequencies of CD4^+^ and CD8^+^ T cells, calculated as a percentage of total cells within the tissue; Frequencies of PD-1, TIGIT, and CD226 expression on CD4^+^ and CD8^+^ T cells, shown as a percentage of the parent T-cell population; and Linear regression analysis showing a significant positive correlation between the density (cells/mm^2^) of TIGIT^+^ and CD226^+^ cells within the CD4^+^ (left) and CD8^+^ (right) T cell populations. R^2^ and P values are shown. **(H)** Correlation analysis between the density of total PD-L1^+^ cells (left) or total CD155^+^ cells (right) and the density of CD4^+^ (red line) or CD8^+^ (blue line) T cells.

Sensitivity to PD-1 blockade has been shown to be determined not only by expression of PD-L1 but also CD155 (PVR) (25, 30, 31). To experimentally validate the hypothesis that the bispecific format of HB0036 confers an advantage by bridging its targets in *cis*, we dissected how the spatial expression of PD-L1 and CD155 impacts the capacity of immune restoration. We used a CRISPR/Cas9 system to generate HT1080 tumor cells expressing both targets (double-positive: CD155^+^PD-L1^+^), single targets (CD155 single-knockout, CD155^KO^: PD-L1^+^CD155^-^ or PD-L1 single-knockout, PD-L1^KO^: PD-L1^-^CD155^+^), or neither (CD155 and PD-L1 dual-knockout, DKO: PD-L1^-^CD155^-^) (**Figure 6A**). As hypothesized, in the presence of wild-type HT1080 cells where both ligands are co-expressed on the same cell, HB0036 demonstrated a clear and significant advantage over the antibody mixture in reversing T-cell immune inhibition (**Figure 6B**), consistent with our earlier results (**Figures 1B, C**). Crucially, this advantage was completely abrogated when PD-L1 and CD155 were provided on separate cells (a mix of PD-L1^+^/CD155^-^ and PD-L1^-^/CD155^+^ cells) (**Figure 6B**). In the absence of CD155, both HB0036 and the antibody mixture mediated similar levels of immune restoration, attributable to PD-L1 blockade alone. In the absence of PD-L1, neither treatment affected T-cell proliferation. These data provide direct functional evidence that the superior efficacy of the HB0036 bispecific antibody is dependent on the co-expression and spatial proximity of its targets on a single cell, a condition that allows for effective co-engagement.

The data suggest that the expression patterns of PD-L1 and CD155 significantly influence T-cell immunity and the efficacy of PD-L1/TIGIT co-blockade. We performed a high-content analysis of the tumor immune microenvironment, focusing on the spatial distribution of PD-L1 and CD155 in lung adenocarcinoma (LUAD) tissue samples. In a cohort of 20 patient samples, high PD-L1 expression was observed in only a small subset of samples. Notably, PD-L1 was more prevalent in the stromal compartment (comprising immune cells and cancer-associated fibroblasts) than on tumor cells themselves (**Figure 6D**). CD155 expression, primarily localized to PanCK^+^ tumor cells, varied considerably among patients (5~80%; **Figure 6E**). A similar trend was observed in an independent dataset of five LUAD samples (**Supplementary Figure 5A**). When assessing co-expression of CD155 and PD-L1 on PanCK^+^ tumor cells, approximately 20% of the cohort exhibited a CD155^+^/PD-L1^+^ phenotype (**Figure 6F**), consistent with prior findings in a larger NSCLC cohort (62% adenocarcinoma) (25). T-cell populations within the tumor microenvironment were also analyzed. Both CD4^+^ and CD8^+^ T cells were present (**Figure 6G**), with subsets expressing the inhibitory receptor TIGIT and the co-stimulatory receptor CD226, alongside low PD-1 levels. Notably, TIGIT^+^ and CD226^+^ cell densities showed a strong positive correlation in both CD4^+^ and CD8^+^ populations (**Figure 6G**). Overall, PD-L1 expression correlated positively with CD4^+^ and CD8^+^ T-cell density, whereas CD155 exhibited a negative trend with CD8^+^ T-cell infiltration (**Figure 6H**).

Beyond patient samples, we evaluated CD155 and PD-L1 mRNA expression in patient-derived xenografts (PDXs) and human cancer cell lines using the CROWN BIOSCIENCE and CCLE databases. CD155 was broadly elevated across cancer types (except hematologic malignancies), while high PD-L1 expression was limited to a minor fraction of tumor cells in most cancers (**Supplementary Figures 5B, C**). PDX analysis revealed that lung cancer had the highest CD155/PD-L1 co-expression frequency (**Supplementary Figure 5D**). Protein-level assessment in selected cell lines further confirmed heterogeneous expression patterns (**Supplementary Figure 5E**). Notably, PD-L1 regulation differs between tumor cells and tumor-infiltrating immune cells, with distinct roles in antitumor immunity (24). While our study primarily focused on tumor-intrinsic PD-L1 in drug accumulation and immune modulation, we also observed PD-L1 upregulation in GM-CSF-stimulated human monocytes (**Supplementary Figure 5F**) and murine tumor-derived myeloid cells (**Supplementary Figure 5G**). These findings underscore the complexity of PD-L1/TIGIT co-blockade mechanisms.

## Discussion

The concept of enhancing anti-tumor immunity by co-blocking PD-L1 and TIGIT is well-established, and several bispecific antibodies (BsAbs) have shown preclinical promise (13, 14). The central question this study addresses is not whether co-blockade is effective, but rather why and under what conditions a bispecific antibody format is superior to the co-administration of two separate antibodies. Our findings provide key mechanistic insights into this question. We demonstrate for the first time, through direct functional experiments (**Figure 6**), that the superior ability of the BsAb HB0036 to reverse T-cell inhibition is critically dependent on the cis-co-expression of PD-L1 and CD155 on the same target cell. Furthermore, we identify a unique advantage of the bispecific format in its ability to upregulate the co-stimulatory receptor CD226 on T cells, an effect not observed with the antibody combination (**Figure 1E**). *In vivo*, this translates to enhanced anti-TIGIT antibody accumulation in PD-L1-positive tumors (**Figure 2**) and improved tumor control associated with favorable immunological changes (**Figure 4**). Collectively, these findings shift the focus from the novelty of the target pair to the mechanistic advantages of the molecular format, providing a strong rationale for developing BsAbs and suggesting that target co-expression patterns are a key biomarker for clinical translation.

Differences between the bispecific antibody and mAb combination are evident in the syngeneic CT26-hPD-L1 tumor model and the BxPC-3 xenograft model. In the syngeneic CT26-hPD-L1 tumor model, tumor control is superior in mice treated with HB0036. Improved tumor control is associated with favorable immunologic characteristics, including higher intratumoral levels of IFN-γ and TNF-α, and a higher proportion of CD8^+^ T cell infiltrates. As CT26-hPD-L1 co-expresses hPD-L1 and mCD155, hPD-1/hPD-L1/hTIGIT humanized mice may potentially express hPD-L1 in host myeloid cells. The contribution of PD-L1 from immune cells remains to be clarified. In the BxPC-3 xenograft model using PBMC humanized mice with few reconstituted human myeloid cells, PD-L1 primarily originates from the grafted BxPC-3 xenograft. A moderate but significant improvement in tumor control with HB0036 is observed. Analysis of intratumoral infiltrates reveals that HB0036-treated tumors have a relatively more abundant host monocytic myeloid cell composition. Specifically, we observed a significant reduction in Ly6G^+^ neutrophils, which often represent granulocytic myeloid-derived suppressor cells (G-MDSCs) associated with T-cell suppression and a concurrent enrichment of Ly6G^-^ myeloid populations in the HB0036 group. This remodeling of the myeloid compartment creates a more permissive immune microenvironment, potentially reducing immunosuppression and synergizing with T-cell reinvigoration to support the superior tumor control observed with HB0036. These findings align with recent clinical observations where enriched monocytic myeloid cells were linked to improved outcomes in TIGIT co-blockade therapies (32). The HB0036 group also shows a marked reduction in donor TIGIT^+^ T cells and an increase in CD226^+^ and CD96^+^ cells. We cannot determine whether this reduction is due to the deletion of TIGIT^+^ cells, TIGIT downregulation, or antibody-induced TIGIT internalization.

Considering both *in vitro* and *in vivo* data, PD-L1 and TIGIT co-blockade using a bispecific antibody may offer advantages over a combination of two mAbs. The potential mechanisms may involve enhanced co-engagement of the PD-L1/PD-1 and CD155/TIGIT/CD226/CD96 axes. For *in vivo* effects, the enhanced accumulation of the bispecific antibody to maintain anti-TIGIT potency could be advantageous over the combination, particularly in PD-L1 high tumors. This may also be true when compared to PD-1 targeting bispecific antibodies.

The depletion of intra-tumoral Treg cells by an Fc-competent anti-TIGIT antibody is recognized as a mechanism contributing to the inhibition of tumor expansion induced by the anti-TIGIT antibody (26). *In vitro*, both anti-TIGIT mAbs, HB0030 and HB0036, effectively kill Treg cells via NK cells, with HB0030 demonstrating superior efficacy. This cytotoxic effect is clearly dependent on intact antibody-dependent cellular cytotoxicity (ADCC), as the ADCC-silent forms of both antibodies do not mediate Treg cell killing. In a tumor setting, HB0036 also leads to a reduction in intratumoral Treg cells within 24 hours following antibody injection. However, subsequent experiments comparing anti-tumor efficacy of HB0036, and combination therapies did not reveal a prominent and selective reduction in Treg cells. We hypothesize that the intratumoral Treg pool is likely influenced by multiple factors, including trafficking and local generation or conversion. Direct killing mediated by the anti-TIGIT antibody is only one facet of TIGIT targeting. Interestingly, a recent study indicated that a higher abundance of Treg cells was associated with improved overall response rates (ORR) in co-blockade regimens compared to those treated with atezolizumab alone (32). Thus, the overall impact of co-blockade on Tregs and their contribution to the immune response remains to be fully elucidated.

Limited enhanced benefits from PD-(L)1 and TIGIT co-blockade has prompted the addition of a known effective target, VEGF, to improve clinical outcomes (20). In preclinical studies, we compared two combination formats: HB0036 with HB002.1T (VEGF trap) (22) and HB0025 with HB0030, in a syngeneic hepatocellular carcinoma (HCC) H22 hPD-L1 model. Both combinations demonstrated enhanced tumor control compared to BsAbs alone. A similar conclusion was drawn from the combination of HB0025 and HB0030 in a syngeneic MC38 hPD-L1 model and EMT6 model. Notably, triple blockade did not result in significant weight loss, indicating reasonable safety. The anti-PD-1/TIGIT bispecific antibody Rilvegostomig has also been trailed with the ADC drug datopotamab deruxtecan for metastatic non-small cell lung cancer (TROPION-Lung10, NCT06357533). Exploring the combination of HB0036 or HB0025 with ADCs could further expand current immunotherapy options.

It is increasingly evident that the efficacy of PD-1/PD-L1 blockade is influenced not only by PD-L1 expression but also by CD155 (PVR) expression (25, 30, 31). PD-L1 expression is intricately regulated on both tumor and immune cells through distinct mechanisms, playing non-redundant roles in anti-cancer immunity (24). Furthermore, our study indicates that PD-L1 and CD155 may exhibit differential spatial distribution. These complexities could impact the determination of patients likely to benefit from HB0036 treatment. If co-expression of PD-L1 and CD155 is essential for HB0036, the determining factor is likely PD-L1 expression, whether on tumor or immune cells, since CD155 is expressed in most cells. Evaluating the expression of CD226, TIGIT, and PD1 may aid in assessing HB0036 efficacy. Overall, a PD-1/PD-L1 and TIGIT co-blockade in the form of a bispecific antibody (BsAb) may have a narrow window to be superior to conventional combination and may require multiply biomarkers to stratify suitable patients.

This study has several limitations that should be acknowledged. First, our *in vivo* conclusions are drawn exclusively from subcutaneous tumor models. These models may not fully recapitulate the complex tumor microenvironment of orthotopic sites, and future studies in such models are warranted to confirm the translational relevance of our findings. Second, while some experiments showed trends toward improved tumor control (e.g., **Figure 4C**), the differences were modest and were not extended to evaluate a potential benefit in median survival, which remains a more definitive endpoint for preclinical efficacy. Furthermore, while improved tumor control was associated with favorable immunological changes, the direct causal link between these alterations in the TME and the therapeutic effect was not formally established. Third, our investigation into the triple blockade with an anti-VEGF agent, while showing enhanced tumor control, did not include mechanistic studies on the tumor vasculature, such as changes in perfusion or vessel normalization, which could explain the observed synergy. Finally, while we demonstrated PD-L1-dependent enrichment of HB0036 in tumors, the precise contribution of PD-L1 expressed on host immune cells versus tumor cells to this effect *in vivo* remains to be fully elucidated. Despite these caveats, this study provides valuable functional evidence for the mechanistic advantages of a bispecific antibody in co-targeting PD-L1 and TIGIT and offers a strong rationale for the clinical investigation of biomarkers based on target co-expression.

## Materials and methods

### Animal and human PBMC cells

Animal experiments were conducted with the approval of the Institutional Animal Care and Use Committee of Gempharmatech Co., Ltd (Approval Nos. GPTAP20220413-2, GPTAP20220624-4, GPTAP20220818-1, GPTAP20201214-3, GPTAP011), Biocytogen Pharmaceutical (Beijing) Co. (Approval No. BAP-BJ-PS-01-2204158), and Pharmalegacy Co. (Approval No. PL220119-3). The tumor cell lines, and mouse strains used in this study are listed in the supplemental materials. All procedures adhered to the principles of laboratory animal welfare and ethics, including the “3R principles.” Humane care was provided to all laboratory animals throughout the research process. For human PBMCs, ethical approval was obtained from the Ethics Committee of Shanghai Zha Xin Integrated Traditional Chinese and Western Medicine Hospital (Approval No. SL-WBC-202001). The antibodies and biological materials used in this study are listed in **Supplementary Table**.

### Generation of monoclonal and bispecific antibodies

*Characterization of Humanized Antibodies:* The humanized anti-PD-L1 monoclonal antibody (WO2020199860A1, NCT04678908) and the humanized anti-TIGIT antibody HB0030 (WO2021227940A1, CTR20212828) have been previously reported. Briefly, murine antibody variable region genes were cloned from monoclonal hybridoma cells targeting the human PD-L1 extracellular domain (ECD) or human TIGIT ECD. These genes were humanized by grafting the complementarity-determining regions (CDRs) into human homologous frameworks. The humanized antibody variable genes were then cloned into expression vectors containing the constant regions of the human IgG1 heavy chain and kappa light chain.

*Preparation of HB0030-ADCC Silent Antibody*: To eliminate binding affinity to Fc receptors and complement component C1, and thus abolish antibody-dependent cell-mediated cytotoxicity (ADCC) and complement-dependent cytotoxicity (CDC) activities, leucines at positions 234 and 235 in the CH2 region of the HB0030 Fc domain were mutated to alanines (L234A/L235A). Plasmids containing the corresponding antibody DNA were transfected into CHO- S or CHO-K1 cells. Antibodies were purified from culture supernatants by ProteinA affinity chromatography.

*Generation of Bispecific Antibody:* The variable region sequences of the parent anti-PD-L1 and anti-TIGIT (HB0030) were generated using hybridoma technology. The humanized anti-TIGIT was selected as the single-chain variable fragment (scFv) component of the IgG-scFv, arranged in a VL VH orientation with a (GGGGS)n (n=3, 4, 5) linker. A disulfide bond was engineered into the anti-TIGIT scFv between the heavy chain variable domain VH44 and the light chain variable domain VL100 to enhance stability. The anti-TIGIT scFv was linked to the C-terminus of the heavy chain of anti-PD-L1 IgG1 Fc using a flexible peptide linker (G4S)4. DNA sequences were synthesized by Genewiz and subcloned into a pcDNA3.1-derived expression vector, amplified in DH5α competent cells. Purified plasmids were transfected into CHO-K1 cells for stable cell line development via the PEI technique, using linearized heavy and light plasmids (total 50 µg, HC: LC = 1:2).

*Purification of BsAb Proteins:* Cell lines were cultured in a shaker for seed expansion until a viable cell density of 0.3×10^6 cells/mL was reached, followed by inoculation in bioreactors for approximately 15 days in fed-batch mode. Antibodies were captured from cell supernatants using protein A resin (MabSelect SuRe, Cytiva) and further purified by anion exchange chromatography (Capto Adhere, Cytiva) and cation exchange chromatography (Poros 50HS, Thermo Fisher).

### Hydrogen-deuterium exchange detected by mass spectrometry

Purified antigen (5 µM), antibody (5 µM), or their pre-formed complex (1:1 molar ratio) were incubated in buffer (50 mM HEPES, pH 7.5, 50 mM NaCl, 5% glycerol, 4 mM MgCl_2_, 2 mM DTT) for 1 hour at 4 °C prior to hydrogen-deuterium exchange (HDX) reactions. For exchange, 4 µL of each sample was mixed with 16 µL of D_2_O-containing exchange buffer (50 mM HEPES, pH 7.5, 50 NaCl, 2 mM DTT) and incubated at 4 °C for varying time points (0, 10, 60, 300, and 900 s). Reactions were quenched by adding 20 µL of ice-cold quenching solution (3 M guanidine HCl, 1% trifluoroacetic acid), and quenched samples were promptly injected into the LEAP Pal 3.0 HDX platform.

Upon injection, samples were digested online using an immobilized pepsin column (2 mm × 2 cm) at a flow rate of 120 µL/min. Resulting peptides were trapped and desalted on a C18 PepMap300 column (Thermo Fisher Scientific), followed by separation on a Hypersil Gold C18 analytical column (2.1 mm × 5 cm, 1.9 µm particles; Thermo Fisher Scientific) with a 6-minute linear gradient (4–40% CH_3_CN, 0.3% formic acid). All steps—sample handling, digestion, and separation—were performed at 4°C to minimize back-exchange. Mass spectrometry data were acquired using a Fusion Orbitrap instrument (Thermo Fisher Scientific) at a resolution of 65, 000 (at *m/z* 400).

HDX experiments were conducted in triplicate using independently prepared antigen-antibody complexes. For each peptide, the intensity-weighted mean *m/z* centroid value was computed and converted to percent deuterium incorporation. Differential HDX data were assessed for statistical significance (*p*-values) via unpaired *t-tests* at each time point, integrated into HDX Workbench software. Back-exchange correction assumed 70% deuterium recovery, accounting for the 80% D_2_O content in the exchange buffer.

To resolve deuterium incorporation at single-residue resolution, data from overlapping peptides were consolidated using a residue-averaging approach. Deuterium levels and peptide lengths were compiled for all overlapping peptides covering each residue. A weighting function prioritized shorter peptides (higher spatial resolution) over longer ones. Weighted averages yielded a single deuterium incorporation value per residue, excluding the first two residues of each peptide and all prolines.

### Surface plasmon resonance binding assay

SPR experiments were performed on a Biacore 8K™ system (Cytiva, Sweden) with the sample compartment and flow cell maintained at 25°C. Data were collected at 10 Hz. Antibodies were captured as ligands on a Protein A sensor chip (Cytiva, Cat#: 29127556) by flowing over the chip surface for 60 seconds at 10 µL/min. HB0036 was diluted in running buffer to achieve optimal capture levels. TIGIT Binding: To assess HB0036’s affinity for human TIGIT, the PD-L1 binding site was first saturated with 200 nM human PD-L1 (ACRO Biosystems, Cat#: PD1-H5229), followed by injection of 20 nM human TIGIT (ACRO Biosystems, Cat: #TIT-H52H5) in 3-fold serial dilutions. PD-L1 Binding: For PD-L1 affinity measurements, TIGIT binding was pre-saturated with 50 nM human TIGIT, followed by injection of 50 nM human PD-L1 (3-fold dilutions). Each analyte was injected for 120 seconds (association phase) and dissociated for 600 seconds at 30 µL/min. Between cycles, the sensor surface was regenerated with 10 mM glycine (pH 1.5) injected for 30 seconds at 60 µL/min.

Binding affinities of the parental antibody (HB0030 PD-L1 WT IgG1, #900458) and control antibodies (#900542 and atezolizumab) were determined identically, omitting enhancement steps. Data were analyzed using single-cycle kinetics (capture analysis mode) in Biacore Insight Evaluation Software, fitting to a 1:1 binding model with local kinetics.

HB0036, HB0030, and #900458 were captured at specified levels (360 RU for HB0036; 250 RU for HB0030 and #900458). Human PD-L1 (200 nM) and TIGIT (50 nM) were injected sequentially (120 seconds each, 10 µL/min), followed by regeneration. The binding stoichiometry was calculated as:


Stoichiometric Ratio=[Analyte][Ligand]Stoichiometric Ratio=[Ligand][Analyte]

where: [Ligand]=Response Units (Capture Level)MWLigand, [Analyte]=Response Units (Analyte Level)MWAnalyte[Ligand]=MWLigand​Response Units (Capture Level)​, [Analyte]=MWAnalyte​Response Units (Analyte Level)​. Molecular weights (MW) were derived from HPLC-verified homodimers (ACRO Biosystems): PD-L1: 26, 000 Da and TIGIT: 28, 800 Da.

### Luciferase reporter gene system assays

Jurkat-NFAT-PD-1-5B8 cells are engineered Jurkat cells stably expressing human PD-1 and an NFAT promoter-driven luciferase reporter. CHO-K1-OS8-PD-L1-8D6 cells are modified CHO-K1 cells co-expressing PD-L1 and the T-cell activator OKT3 scFv, which stimulates Jurkat cells to induce NFAT-mediated luciferase expression. For the assay, Jurkat-NFAT-PD-1-5B8 and CHO-K1-OS8-PD-L1-8D6 cells were seeded at 5 × 10^5^ cells/mL (30 µL/well) in a 96-well plate. Serially diluted antibodies (30 µL/well) were added, followed by incubation at 37 °C with 5% CO_2_ for 6 hours. T-cell activation was quantified using the *Bio-Glo™* luciferase assay system (Promega, Madison, WI) per the manufacturer’s protocol. Relative luminescence units (RLU) were measured on an *MD SpectraMax® i3x* microplate reader (luminescence mode). Dose-response curves (RLU vs. antibody concentration) were analyzed by four-parameter logistic regression in GraphPad Prism to determine EC_50_ values.

### IL-2 production assay

Jurkat-TIGIT-22G8 cells are engineered Jurkat cells stably expressing human TIGIT and producing IL-2 upon stimulation with PHA-M. For the assay, cells were seeded at 4 × 10^6^ cells/mL (50 µL/well) in a 96-well plate. Cells were activated with PHA-M (Yeasen, Cat#: 40110ES08, lot: P2107890; 0.95 µg/mL final concentration) and co-treated with soluble CD155 (Sino Biological, Cat#10109-H02H; 8 µg/mL final concentration). Serially diluted antibodies (50 µL/well) were added, followed by incubation at 37 °C, 5% CO_2_ for 22 hours. IL-2 secretion was quantified using an IL-2 ELISA kit (BioLegend, Cat#: 431801). Absorbance was measured at 450 nm/570 nm using a microplate reader. Dose-response curves (OD450/OD570 vs. antibody concentration) were analyzed by four-parameter logistic regression (GraphPad Prism) to determine EC50values.

### Killing of human TIGIT-expressing jurkat cells by NK cells

Human NK cells were seeded at a density of 2×10^6^ cells/mL in a 24-well plate (NUNC) and pre-activated overnight with IL-15 (10 ng/mL, R&D/247-ILB-025). Target cells consisted of CellTrace Violet (CTV)-labeled Jurkat cells expressing human TIGIT. For the killing assay, 10^5^ target cells/well were plated in 50 µL in a 96-well plate. Serially diluted HB0036, HB0030, or isotype controls were added in 50 µL, followed by washed NK cells (1×10^5^/well in 50 µL). After a 5-hour incubation, cells were harvested and stained on ice for 30 minutes with the following antibodies: BV510 anti-human CD56 (BioLegend, Cat#: 318340), APC/Cy7 anti-human CD16 (BioLegend, Cat#: 302017) andFITC anti-human CD107a (BioLegend, Cat#: 328606). Flow cytometry was performed using a BD Canto II with Propidium Iodide (PI, Sigma, Cat#: P4170) for viability assessment. % Cell lysis and integrated MFI (iMFI) were calculated as described. **Killing of Human Tregs by NK Cells**. Tregs were harvested, washed, and labeled with CellTrace Violet (CTV, 1:2000, Thermo Fisher Scientific, Cat#: C34557) for discrimination. 5×10^4^ Tregs/well were seeded in a 96-well round-bottom plate and treated with: Isotype control (wild-type Fc), HB0030, HB0036, HB0030 KO (ADCC-silent) and HB0036 KO (ADCC-silent). NK cells (5×10^4^/well) were added, and co-cultures were incubated overnight. Harvested cells were stained with: PE anti-human CD56 (BioLegend, Cat#: 318306), FITC anti-human CD16 (BD, Cat#: 555406), APC anti-human TIGIT (BioLegend, Cat#: 372706), APC/Cy7 anti-human CD107a (BioLegend, Cat#: 328630). After PI staining, samples were analyzed by flow cytometry.

### Evaluation of tumor inhibition *in vivo*

Two distinct models were employed to assess antitumor efficacy. In all models, treatments were initiated only after tumors were well-established (typically 7–14 days post-inoculation when tumor volumes reached the indicated sizes), ensuring that therapies were administered to established, vascularized tumors.

### Human cancer cells in PBMC-humanized mice

BXPC-3 human pancreatic adenocarcinoma cells (1×10^7^) suspended in a 1:1 mix with Matrigel were injected subcutaneously (200 µL) into the right flank of Prkdc^scid^/Il2rg^null^ (NPG) mice. When tumors reached a volume of 50–80 mm³, mice were randomized (*n=6*/group) and injected intravenously with 1×10^7^ human PBMCs (100 µL). Antibodies were administered intraperitoneally twice weekly for 4 weeks, starting on the day of PBMC injection.

### Mouse cancer cells in target-humanized mice

Model A: HB0030 ± Anti-PD-1 Evaluation: CT26 cells were implanted subcutaneously in BALB/c-hPD-1/hTIGIT mice. Antibody dosing began when tumors reached 60–80 mm³. Model B: HB0030 + Anti-PD-L1 (900458) Evaluation: CT26-hPD-L1 cells (5×10^5^) were implanted subcutaneously in hTIGIT/hPD-L1/hPD-1 BALB/c mice. Dosing started at a mean tumor volume of 70 mm³.

For investigation of tumor inhibition with the combination of HB0036 and HB002.1T, or the combination of HB0025 and HB0030, two additional models were used. In one model, hPD1/hPDL1/hTIGIT BALB/c mice were subcutaneously inoculated with H22-hPDL1 cells. Once the average tumor volume reached approximately 100 mm³, mice were randomly grouped with a tumor volume coefficient of variation (CV) ≤ 1/3. Treatment was initiated on day D0, with intraperitoneal administration twice weekly at a volume of 10 μL/g multiplied by the mouse’s body weight (g). For the combination treatment group, each drug was administered separately at double the concentration and half the volume, with a 30-minute interval between administrations. Tumor size and mouse body weight were monitored throughout, with the endpoint being the measurement of tumor weight. In another model, 5×10^5^ MC38/hPD-L1 cells were inoculated subcutaneously on the backs of hTIGIT/hPD-L1/hPD-1 humanized C57BL/6 mice. Tumor volumes (TV) and body weights were measured and recorded throughout the study. At the end, tumor weights were measured and recorded. Some experiments also included analysis of intratumoral infiltrating leukocytes.

### Antibody accumulation at tumor sites in the MC38 tumor model

hTIGIT/hPD-L1/hPD-1 C57BL/6 mice were subcutaneously inoculated with 1×10^6^ wildtype MC38 cells on the left flank near the armpit, and 1×10^6^ hPD-L1^+^ MC38 cells on the right flank. Once tumors reached approximately 200 mm³, mice were administered intraperitoneally with one of the following: 683 µg of biotinylated bispecific HB0036 (at an equimolar concentration to other groups), 500 µg of anti-TIGIT HB0030, 500 µg of anti-PD-L1 900458, a combination of 500 µg HB0030 and 500 µg 900458, 500 µg of Control Ig 900201, or PBS. Tumors were harvested 24 hours post-injection. Tumor cells and immune infiltrates from tumor digests were analyzed for antibody accumulation and phenotypic characterization. For the flow cytometry (FACS) assay, cells were incubated on ice with fixable Zombie Violet dye (BioLegend, Cat#: 423114) and His-tagged TIGIT (ACRO Biosystems, Cat#: TIT-H52H5). Subsequently, cells were stained with PE-Streptavidin (BioLegend, Cat#: 405204), BV510 anti-mouse CD45 (BioLegend, Cat#: 103138), APC/CY7 anti-mouse CD4 (BD, Cat#: 565650), AF488 anti-mouse FoxP3 (BioLegend, Cat#: 320012), and PE/CY7 anti-human TIGIT (BioLegend, Cat#: 372714). The binding of anti-TIGIT antibodies (HB0036/HB0030) to the cell surface was detected using APC-anti-His (BioLegend, Cat#: 362605). For the ELISA assay of anti-TIGIT antibodies in tumor digests, recombinant TIGIT-His protein was coated onto 96-well plates and incubated overnight at 4 °C. Tumor digests were treated with 2% Tween-20 (Sangon, Cat#: A100777) to extract proteins. The extracts were then transferred to the TIGIT-His coated plates and incubated for 2 hours, followed by three washes. Detection was performed with streptavidin-HRP (CST, Cat#: 3999S), and plates were washed six times. TMB substrate (BioLegend, Cat#: 421101) was added, followed by stop solution. Absorbance was measured at 450 nm (OD450).

### Deletion of PD-L1 and CD155 of tumour cells

sgRNAs (PD-L1: 5’TCTTTATATTCATGACCTAC; CD155: 5’CCCGAGCCA TGGCCGCCGCG) were chemically synthesized (GenScript). Ribonucleoproteins (RNPs) were produced by complexing sgRNA and recombinant spCas9 (Sino Biological) for 10 min at room temperature. HT1080 cells were mixed with RNPs and subjected to electroporation immediately after complex formation. Harvested cells were then stained for surface PD- L1 and CD155. Four cell populations (PD-L1^+^CD155^-^, PD-L1^-^CD155^+^, PD-L1^+^CD155^+^, PD-L1^-^CD155^-^) were sorted by flow cytometer and cultured for *in vitro* study.

### *In vitro* evaluation of T-cell stimulation with anti-PD-L1 and TIGIT Abs

HT1080, and CD155 KO, PD-L1 KO, dual-KO HT1080 was harvested and incubated with mitomycin (10 g/mL) at 37°C for 30 min. HT1080 was seeded in 96-well U bottom plate at 2×10^4^/well after washing and incubated with fresh media overnight. Plates after wash were then incubated with antibodies HB0036, Combination (HB0030 + 900458), HB0030, 900458, 900201. Human PBMCs labelled with CTV was then added at 1×10^5^ cells/well in media with cocktail of anti-CD3, anti-CD28 antibodies and IL-7. After 4–5 days’ incubation, the cells were then subjected to FACS analysis for activation of T and NK cells while the culture supernatants were evaluated for cytokines. The expression of CD226, CD96, TIGIT, PD-1 in this system was evaluated. In the system without HT1080, soluble CD155 and PD-L1 was incubated with PBMCs. In some cultures, ADCC-silent anti-TIGIT/PD-L1 antibody 700001 and anti-TIGIT antibody were also included.

### Analysis of tumour infiltrated leukocytes

Tumours were grinded and digested with gentle MACS Octo Dissociator in the presence of DNase. Harvested tumour digests were filtered through 70 m before centrifuging at 800 g for 10 min for cell pellet and resuspended in volumes adjusted according to tumour mass. Then cell number were counted. Leukocytes of human and mouse origin were analysed. For spontaneous cytokine release, tumour digests (2×10^5^ cells) were cultured in 96-well plate for 24 hours. Cytokines were evaluated by either ELISA or Cytometric Bead Array.

### Analysis of expression PD-L1, CD155 and related molecules

Data on mRNA expression of PDL1, CD155 and other molecules by PDX and cell lines were downloaded from CROWN BIOSCIENCE (*https://www.crownbio.com/databases*) and CCLE (*https://depmap.org/portal/*). Data were classified based on tumour type. Scatter plots with mean and SD were analyzed by GraphPad Prism8.0.1, and each dot indicated data from one PDX or cell line. Correlation between PDL1 and CD155 of several types of tumor were plotted by the same soft. For surface expression by cancer cell lines, cells were stained with indicated antibody. Gated live cells were shown for expression of PD-L1 and CD155.

### Analysis of expression PD-L1, CD155 and related molecules of patient samples

Two cohort of patient samples were analyzed with different platforms. For a large cohort (*n=20*), the protocol was described as following: Multiplex Immunohistochemistry (mIHC) Staining Formalin-fixed, paraffin-embedded (FFPE) tissue sections were stained using a sequential mIHC protocol based on tyramide signal amplification (TSA) technology on an automated stainer (QPath 48, C-HP-88). Briefly, the workflow for each cycle included deparaffinization and rehydration, followed by heat-induced epitope retrieval (HIER). Endogenous peroxidase activity was blocked with 0.3% H_2_O_2_ for 10 min at room temperature. Sections were then sequentially incubated with a primary antibody, a signal amplifier (AMP), a horseradish peroxidase (HRP)-conjugated secondary antibody polymer, and the corresponding fluorophore. The HIER step also served to strip antibody complexes from the preceding cycle. Two distinct antibody panels were used in this study: Panel 1 (5-plex): This panel was applied in the following sequence: 1st, PTK7 (1:1000, pH 6.0 HIER) with CY5 (1:100); 2nd, CD155 (1:400, pH 9.0 HIER) with XFD488 (1:200); 3rd, PD-L1 (1:2400, pH 9.0 HIER) with XFD594 (1:400); 4th, CD73 (1:4000, pH 9.0 HIER) with CY3 (1:100); and 5th, PanCK (1:1200, pH 9.0 HIER) with CF680R (1:200). All primary antibody incubations were performed for 60 min at room temperature. Panel 2 (6-plex): For this panel, HIER was performed in a pH 9.0 buffer for all cycles. The staining order was as follows: 1st, PD-1 (1:300) with XFD594 (1:400); 2nd, following a 30 min blocking step with 10% goat serum, sections were incubated with TIGIT antibody (1:1200) overnight at 4 °C, followed by detection with CY3 (1:100); 3rd, CD8 (1:240) with CF430 (1:200); 4th, CD4 (1:900) with XFD488 (1:200); 5th, CD226 (1:2000) with CY5 (1:100); and 6th, PanCK (1:1200) with XTSA690 (1:600). With the exception of TIGIT, all primary antibody incubations were performed for 60 min at room temperature. Upon completion of all staining cycles, slides were counterstained with DAPI to visualize cell nuclei and were coverslipped using an anti-fade mounting medium. Image Acquisition and Analysis. Whole-slide images were acquired using a digital pathology scanner (KF-FL-020, B-PD-23) for subsequent analysis.

For a smaller cohort (*n=5*), following protocol was used. Tissue slides underwent de-paraffinization and rehydration through xylene and a graded ethanol series, followed by washes in distilled water and fixation in formalin. Antigen retrieval was performed using a microwave and antigen retrieval solution (Panovue). Slides were then blocked, incubated with primary antibodies: anti- PanCK (Sigma, Cat#: C2562), anti-PD-L1 (CST, Cat#: 13684), anti-CD155 (CST, Cat#: 81254S), anti- TIGIT (CST, Cat#: 99567S), anti-CD226 (CST, Cat#: 66631), anti-PD-1 (CST, Cat#: 86163), anti-CD4 (ZSGB-Bio, Cat#: ZM0418), anti-CD8 (CST, Cat#: 70306), and HRP-secondary antibodies (Panovue), and subjected to signal amplification using Diaminobenzidine (Biolynx). A second antigen retrieval step was performed after signal amplification. Finally, slides were counterstained with DAPI, mounted, and coverslipped. Stained tissue sections were examined using an Olympus VS200 MTL fluorescence microscope with Olympus UPLXAPO20X objectives. Pathology analysis was conducted using QuPath analysis software on PanoATLAS workstation. Through tissue segmentation, cell segmentation, thresholding, and data summarization, the images were analyzed to generate quantitative data.

### Statistical analyses

The results are presented as mean ± standard error of the mean (SEM). *p* < 0.05 was considered to indicate statistical significance (*p < 0.05, **p < 0.01, and ***p < 0.001 using the Graphpad Prism 9 one-way ANOVA, followed by the least significant difference multiple comparison test). The differences in the TVs between the compared groups were analyzed by two-way ANOVA.

## Data Availability

The original contributions presented in the study are included in the article/**Supplementary Material**. Further inquiries can be directed to the corresponding authors.

## Supplementary material

Supplementary Figure 1 Generation and epitope characterization, binding and blocking activity of the tetravalent bispecific antibody HB0036. **(A)** Construction of the PD-L1/TIGIT bispecific antibody: The PD-L1/TIGIT bispecific antibody was engineered by fusing a single-chain variable fragment (scFv) from the heavy and light chains of the anti-TIGIT antibody (CTR20212828, patent No. 202010387630.4) to the C-terminal domain of the anti-PD-L1 (NCT04678908) IgG backbone. Both linker 1 and linker 2 sequences were (G4S)4 (upper panel). To enhance stability, an inter-chain disulfide bond was engineered between Q100 of the light chain and G44 of the heavy chain. The anti-TIGIT scFv structure was predicted using the SAbPred tool and depicted in cartoon format, with the anti-TIGIT VH domain in green and the VL domain in cyan (lower panel). **(B)** Binding epitope of the anti-TIGIT Ab: Sequence alignments of TIGIT from various species were performed using ClustalOmega, with secondary structures annotated using ESPript. TIGIT residues involved in binding with HB0036 are marked with yellow boxes, and those involved in binding with CD155 are marked with blue stars (left panel). The HB0036 epitopes on TIGIT were identified by hydrogen-deuterium exchange (HDX) and mapped onto the CD155-TIGIT complex structure (PDB code: 3UDW) using PyMOL. The CD155 cartoon structure is in green, and the TIGIT surface structure is in grey. Binding surfaces of CD155, HB0036, and their overlap are shown in blue, magenta, and yellow, respectively (right panel). **(C)**. Binding epitope of the anti-PD-L1 Ab: Sequence conservation and comparison of PD-L1 from various species were conducted, with secondary structures similarly annotated. PD-L1 residues involved in binding to HB0036 are marked with yellow boxes, and those binding to PD-1 are marked with blue stars (left panel). HB0036 epitopes on PD-L1 were identified by HDX and mapped onto the PD-L1-PD-1 complex structure (PDB code: 3BIK) using PyMOL. The PD-1 cartoon structure is in cyan, and the PD-L1 surface structure is in grey. Binding surfaces of PD-1, HB0036, and their overlap are shown in blue, magenta, and yellow, respectively (right panel). **(D–F)** HB0036 competitive binding activity. (D) HB0036 (blue) binds in tandem to PD-L1 and TIGIT, whereas HB0030 (yellow) and 900542 (brown) bind TIGIT but not PD-L1. (E). HB0036 (blue) binds in tandem to TIGIT and PD-L1, while 900458 (green) and Atezolizumab (violet) bind PD-L1 but not TIGIT. (F) Kinetic sensorgrams of antibody interaction with human PD-L1. Evaluations were performed for HB0036, the HB0036 complex pre-bound to TIGIT, 900458, and Atezolizumab [left]; Kinetic sensorgrams of antibody interaction with human TIGIT. Evaluations were performed for HB0036, the HB0036 complex pre-bound to human PD-L1, HB0030, and 900542 [right]. **(G)** Enhancement of NFAT activation by PD-L1 targeting. CHO-K1-OS8-PD-L1-8D6 cells, expressing human PD-L1 and T-cell activator OKT3 ScFv (OS8), were co-cultured with Jurkat-NFAT-PD-1-5B8 cells expressing human PD-1 and a luciferase reporter driven by an NFAT-RE for 6 hours in the absence of HB0036, 900458, or Atezolizumab. NFAT activation was assessed by measuring luciferase activity (bioluminescence) in the supernatant using the Bio-Glo™ luciferase assay system. **(H**) Enhancement of IFN-γ production by PD-L1 targeting. CD4^+^ T cells from one healthy donor were co-incubated with monocyte-derived DCs from another healthy donor for 5 days, with or without HB0036, anti-PD-L1 Ab 900458, or Atezolizumab. IFN-γ levels in the culture supernatants were evaluated by ELISA. **(I)** Enhancement of IL-2 production by TIGIT targeting. Jurkat-TIGIT-22G8 cells expressing human TIGIT were stimulated with 1 μg/ml PHA-M in the presence or absence of soluble human CD155, with gradient concentrations of HB0036, anti-TIGIT Ab HB0030, and isotype control 900201 for 22 hours. IL-2 levels in the supernatants were assayed using ELISA.

Supplementary Figure 2 (related to **Figure 1**). HB0036 induces a stronger proliferative T cell response. **(A)** The line graphs illustrate the recovery of proliferating cells following treatment with serially diluted antibodies, while the histograms show cell division in response to either serially diluted HB0036 or a combination of anti-TIGIT (HB0030) and anti-PD-L1 (900458) antibodies. **(B**) Representative FACS plots depict the proportion of IFN-γ^+^ cells within gated CD4^+^ and CD8^+^ T cells, with bar graphs summarizing the IFN-γ^+^ cell percentages across different culture conditions (*p* < 0.01, one-way ANOVA). **(C)** FACS plots demonstrate CD226 expression on gated CD4^+^ and CD8^+^ T cells. **(D)** Bar graphs summarize the proportion of IFN-γ^+^ cells among gated CD56^+^ NK cells under various culture conditions (*p* < 0.01, one-way ANOVA). **(E)**. Representative FACS plots display CD107a expression by NK cells in the presence of different antibodies.

Supplementary Figure 3 **(A, B)** (Related to **Figure 3****): (A)** Anti-TIGIT mAb HB0030 inhibits tumor expansion. BALB/c-hPD1/hTIGIT mice were inoculated with 5×10^5^ CT26 tumor cells. Treatment began when tumors reached an average volume of 60 mm³. Mice were administered either vehicle control, HB0030, or an ADCC-silenced variant of HB0030 (3 mg/kg; *n=6*). **(B)** TIGIT/PD-L1/PD-1 blockade modulates CD4^+^ T-cell infiltration. C57BL/6-hTIGIT/hPD-L1/hPD-1 mice were inoculated with hPD-L1^+^ and hPD-L1^−^ MC38 cells bilaterally. After 1 day of treatment with biotinylated antibodies, mCD45^+^ tumor-infiltrating cells were analyzed for CD4^+^ T-cell populations. Data represent mean percentages ± SEM. **(C, D)** (Related to **Figure 4****): (C)** Anti-PD-L1 mAb 900458 suppresses tumor growth. BALB/c-hPD1/hPD-L1 mice were subcutaneously inoculated with 5×10^5^ CT26-hPD-L1 cells. Treatment (vehicle, 900458, or atezolizumab; 3 mg/kg; *n=8*) was initiated at a mean tumor volume of 60 mm³. **(D)** Combined HB0030 and anti-PD-L1 therapy enhances tumor inhibition. C57BL/6-hPD-1/hPD-L1/hTIGIT mice bearing MC38-hPD-L1 tumors (mean volume: 100 mm³) were treated with control (900201), HB0030, 900458, or HB0030 + 900458 (all at 3 mg/kg; *n=6*).

Supplementary Figure 4 (related to **Figure 4**). HB0036 modulates tumor immune infiltration in the hPD-L1 CT26 model. **(A, B)** Tumor-infiltrating leukocytes (TILs) and CD8^+^ TILs (normalized by tumor weight) were increased in HB0036 and combination groups versus controls, with no significant differences between treatments. **(C, D)** CD4^+^ T cell frequencies were modestly reduced in the HB0036 group, without selective depletion of regulatory T cells (Tregs). **(E)** CD226^+^ T cells were slightly elevated in HB0036 and combination-treated mice. **(F)** Myeloid compartment composition remained unchanged across groups. **(G)** Enhanced tumor inhibition was observed in PBMC-humanized mice bearing HT1367 human bladder carcinoma xenografts. **(H)** (related to **Figure 5A**). H22-hPD-L1 cells were subcutaneously implanted into the right flank of BALB/c-hPD-1/hPD-L1/hTIGIT mice. When tumors reached ~100 mm³, mice were randomized and treated with antibodies (*i.p.*, twice per week, 4 weeks). Graphs show body weights of different groups.

Supplementary Figure 5 (related to **Figure 6**). **(A)** Expression of CD155 and PD-L1 in a separate cohort (*n=5*) of human lung adenocarcinoma samples. Representative high-content analysis images of two samples are shown. Quantification depicts the percentage of CD155^+^ and PD-L1^+^ cells within PanCK^+^ (tumor) and PanCK^−^ (stromal) compartments. Lines connect paired tumor and stroma samples from the same patient. **(B–D)** Expression patterns of PD-L1 and CD155 in tumor PDX models **(B)** and cell lines **(C)**. mRNA levels of CD155 and PD-L1 in cancer PDXs and cell lines were analyzed (*data source: CrownBio MuBase*). **(B)** Co-expression of PD-L1 and CD155 in PDX samples. **(E)** Surface expression of CD155 and PD-L1 in selected cell lines detected by flow cytometry. **(F)** PD-L1 expression by adherent cells of GM-CSF**-**stimulated human PBMCs. **(G)** PD-L1 expression by freshly-isolated intratumoral CD11b^+^ cells from mice inoculated with MC38.
