# Supplementary figures and images for: Enhancing anti-tumor immunity through co-blocking PD-L1 and TIGIT by facilitating tumor-directed responses and additional VEGF inhibition
Source: Front Immunol. 2026 Jan 14;16:1746155. doi: 10.3389/fimmu.2025.1746155 (PMC12847297; doi:10.3389/fimmu.2025.1746155)

Figure S1

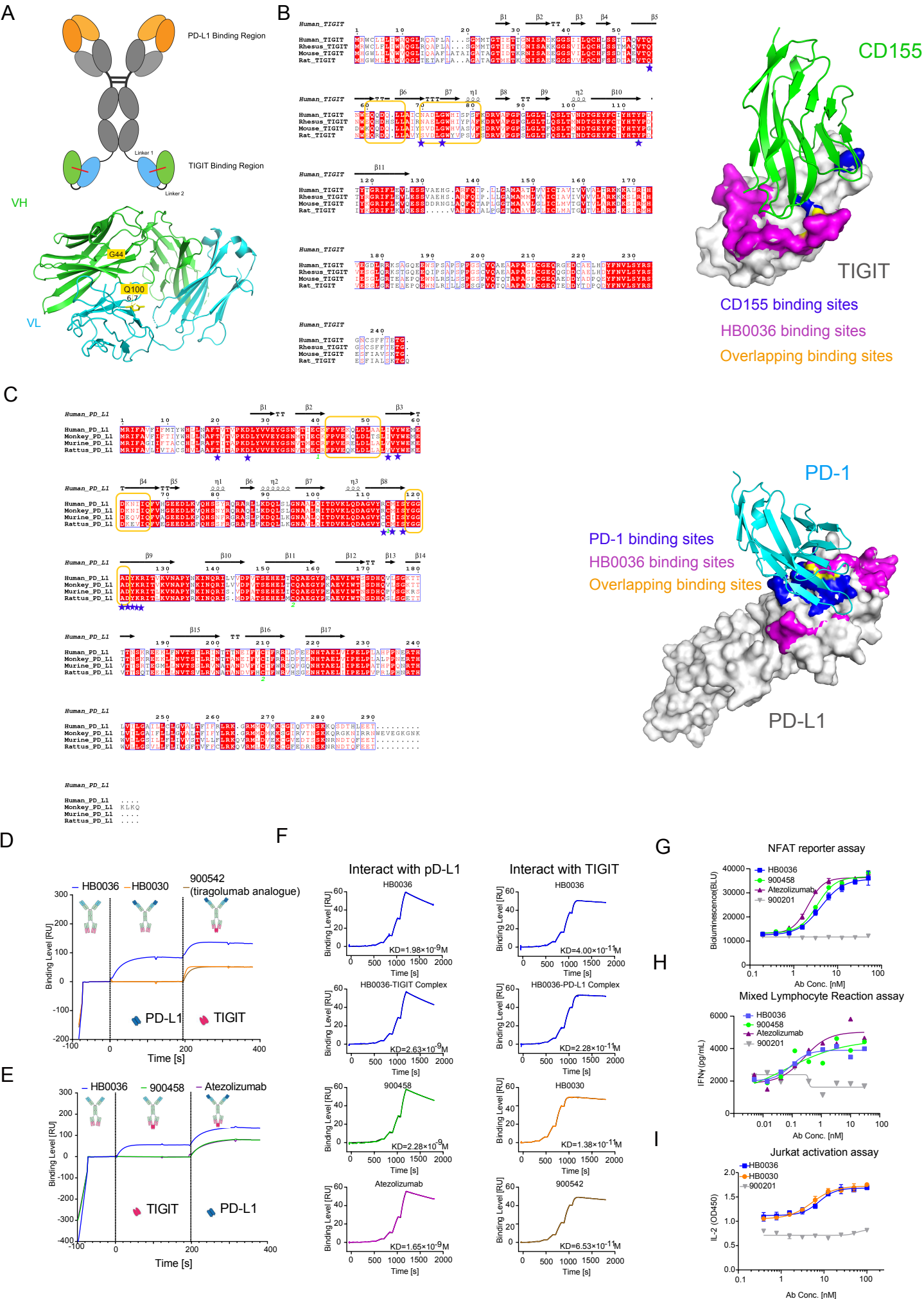

Figure S2

A

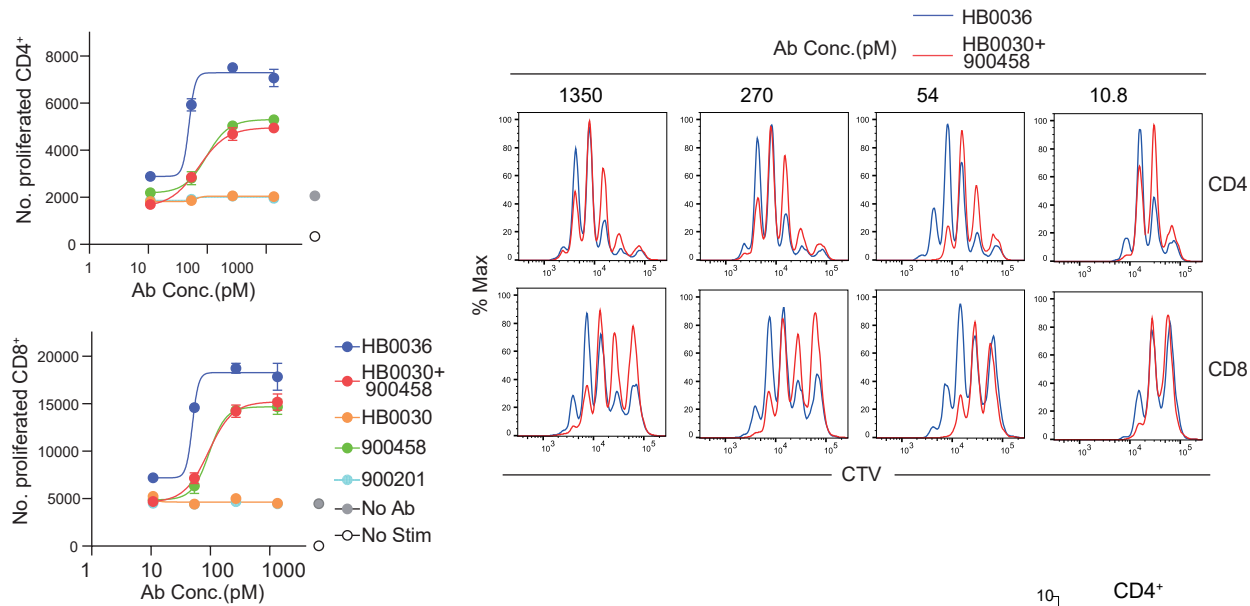

B

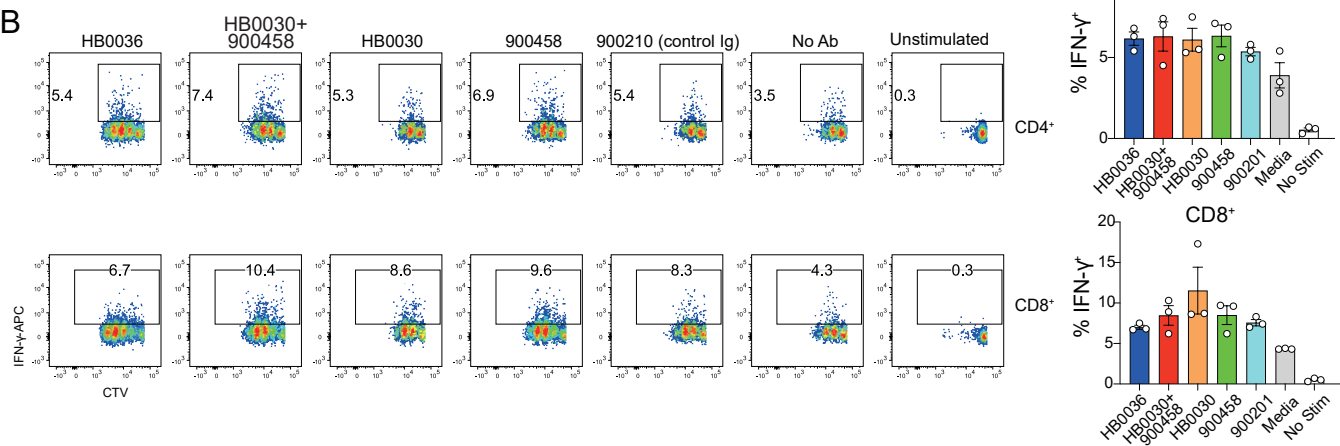

C

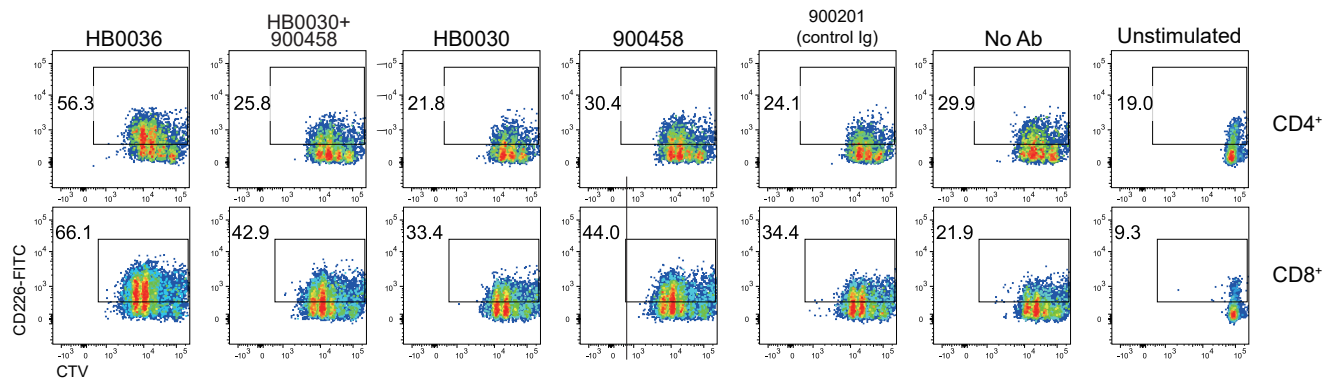

D

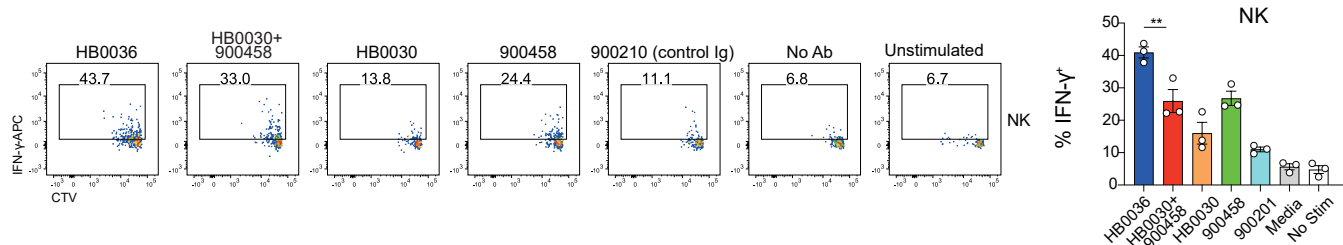

E

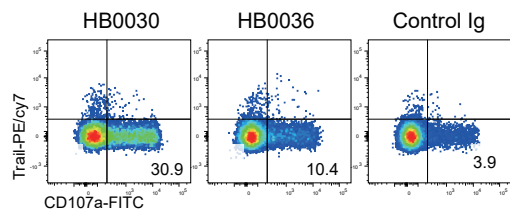

Figure S3

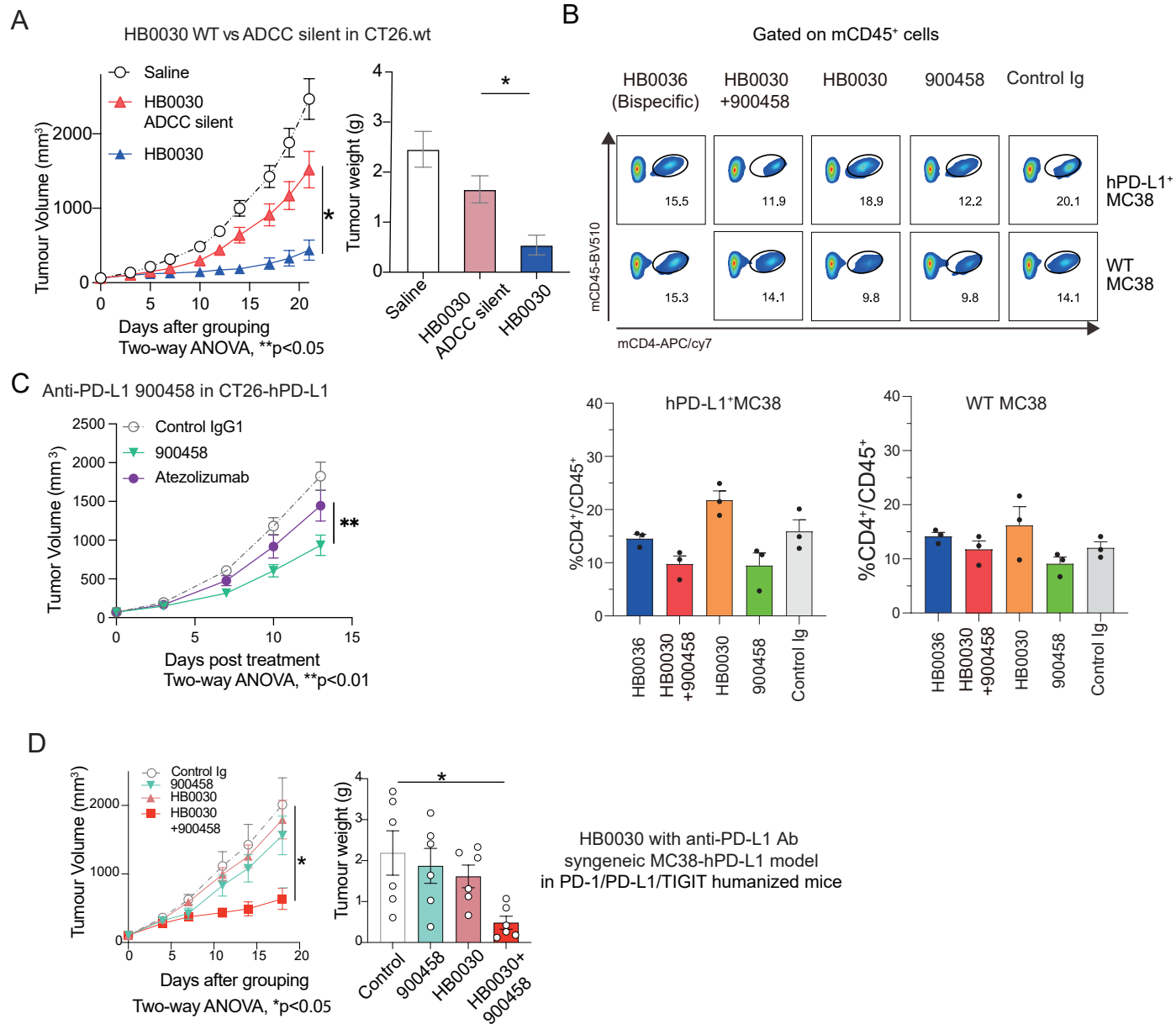

Figure S4

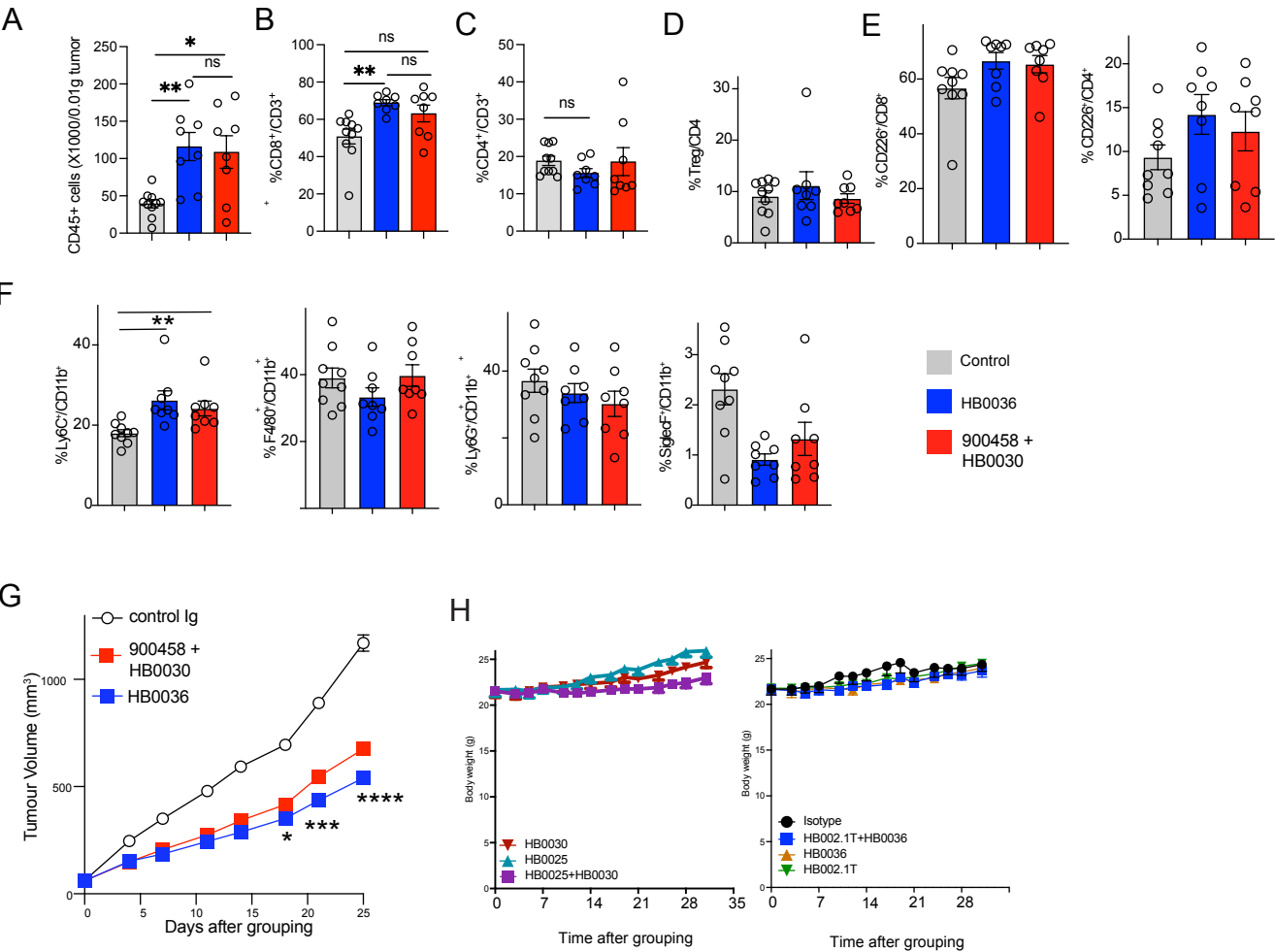

Figure S5

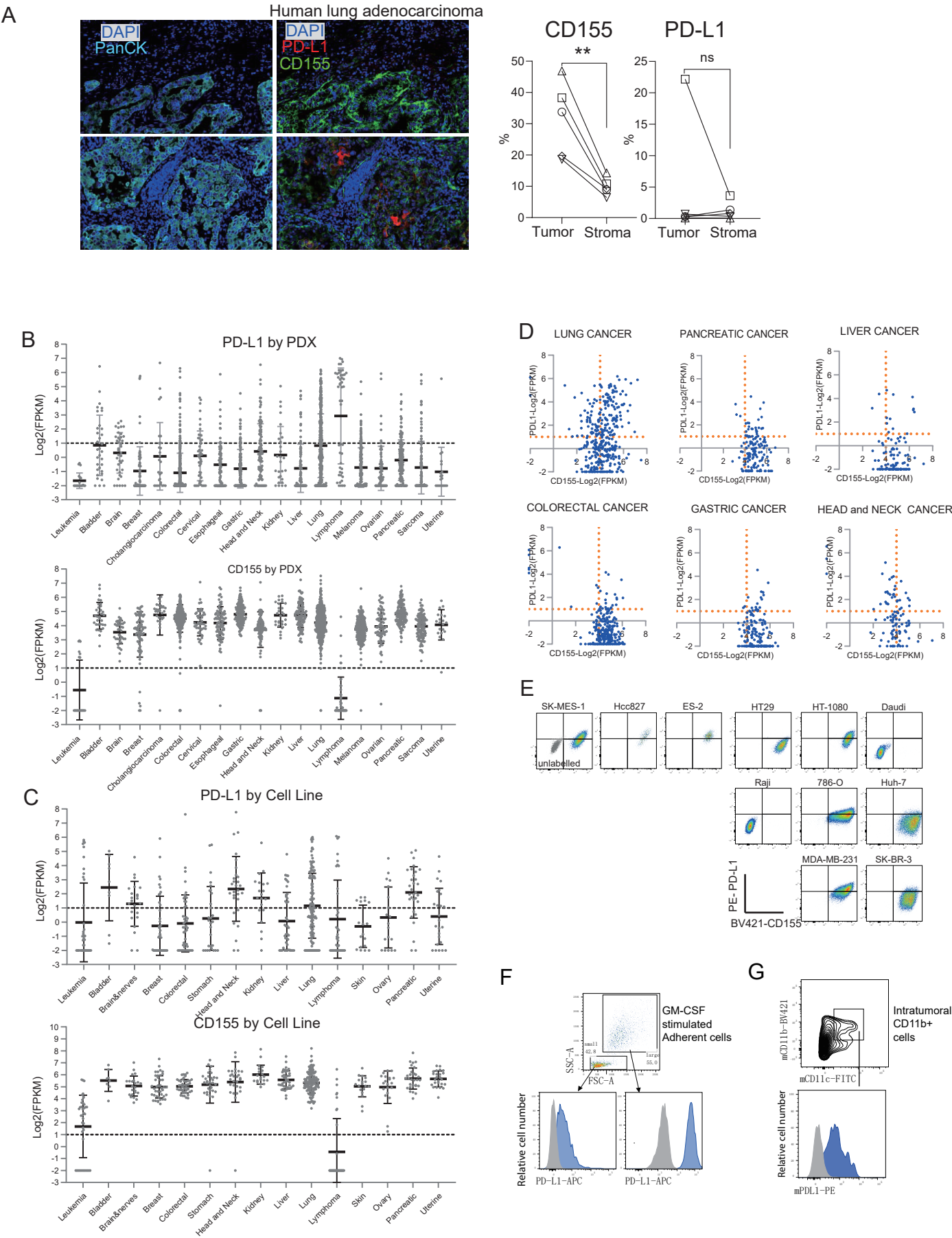

Supplement: Supplementary Figure 1 — Generation and epitope characterization, binding and blocking activity of the tetravalent bispecific antibody HB0036. (A) Construction of the PD-L1/TIGIT bispecific antibody: The PD-L1/TIGIT bispecific antibody was engineered by fusing a single-chain variable fragment (scFv) from the heavy and light chains of the anti-TIGIT antibody (CTR20212828, patent No. 202010387630.4) to the C-terminal domain of the anti-PD-L1 (NCT04678908) IgG backbone. Both linker 1 and linker 2 sequences were (G4S)4 (upper panel). To enhance stability, an inter-chain disulfide bond was engineered between Q100 of the light chain and G44 of the heavy chain. The anti-TIGIT scFv structure was predicted using the SAbPred tool and depicted in cartoon format, with the anti-TIGIT VH domain in green and the VL domain in cyan (lower panel). (B) Binding epitope of the anti-TIGIT Ab: Sequence alignments of TIGIT from various species were performed using ClustalOmega, with secondary structures annotated using ESPript. TIGIT residues involved in binding with HB0036 are marked with yellow boxes, and those involved in binding with CD155 are marked with blue stars (left panel). The HB0036 epitopes on TIGIT were identified by hydrogen-deuterium exchange (HDX) and mapped onto the CD155-TIGIT complex structure (PDB code: 3UDW) using PyMOL. The CD155 cartoon structure is in green, and the TIGIT surface structure is in grey. Binding surfaces of CD155, HB0036, and their overlap are shown in blue, magenta, and yellow, respectively (right panel). (C). Binding epitope of the anti-PD-L1 Ab: Sequence conservation and comparison of PD-L1 from various species were conducted, with secondary structures similarly annotated. PD-L1 residues involved in binding to HB0036 are marked with yellow boxes, and those binding to PD-1 are marked with blue stars (left panel). HB0036 epitopes on PD-L1 were identified by HDX and mapped onto the PD-L1-PD-1 complex structure (PDB code: 3BIK) using PyMOL. The PD-1 cartoon structure is in cya [file DataSheet2.pdf]
